# A multifunctional hydrogel for obesity-associated tumor immunotherapy and postsurgical wound healing promotion

**DOI:** 10.1016/j.bioactmat.2025.11.027

**Published:** 2025-11-26

**Authors:** Hongjuan Zhao, Di Meng, Yajing Wang, Yinke Wang, Qing Li, Yuxin Guo, Qingling Song, Lei Wang

**Affiliations:** a School of Pharmaceutical Sciences, Zhengzhou University, 100 Science Avenue, Zhengzhou, 450001, China; b Key Laboratory of Nanomedicine for Targeting Diagnosis and Treatment, Ministry of Education of China, Zhengzhou University, 100 Science Avenue, Zhengzhou, 450001, China; c School of Pharmaceutical Sciences, State Key Laboratory of Antiviral Drugs, Pingyuan Laboratory, Zhengzhou University, Zhengzhou, 450001, China

**Keywords:** Obesity, T cell immunity, Immunometabolism, Hydrogel, Wound healing

## Abstract

The multidimensional complexity between metabolism and inflammation within the obese tumor microenvironment (OTME) poses substantial barriers to postsurgical immunotherapy and wound management. Herein, we engineered a multifunctional hydrogel (Lipo/CXB@Hydrogel) through covalent conjugation of dopamine-crosslinked oxidized hyaluronic acid and a ROS-sensitive linker, co-delivering the non-steroidal anti-inflammatory drug celecoxib (CXB) and the lipid metabolism modulator Lipofermata (Lipo) to facilitate T cell immunotherapy and wound healing. The unique multi-dynamic-bond crosslinked structure endows the hydrogel with excellent self-healing, tissue adhesiveness and mechanical properties. The implanted Lipo/CXB@Hydrogel degrades and releases CXB to suppress hyperinflammation and enhance intratumoral cytotoxic T lymphocyte (CTL) infiltration, while Lipo inhibits the predatory uptake of fatty acids by tumor cells in the OTME for competing metabolic resources of intratumoral infiltrated CTLs. Importantly, such a cascaded immunological effect of Lipo/CXB@Hydrogel treatment amplifies CTL proliferation and activity specifically through targeting the arachidonic acid (AA)/COX-2/PGE2 signaling axis, a central hub linking lipid metabolism and inflammation, initiating a long-lasting immune response to suppress colorectal tumor postsurgical recurrence and metastasis in obesity contexts. Moreover, the hydrogel can easily repeatedly close the reopened wounds and promote skin regeneration. Thus, this multifunctional hydrogel may provide a promising strategy for postsurgical obese tumor immunotherapy and wound closure.

## Highlights

• **Multifunctional Hydrogel Platform:** A multi-dynamic-bond crosslinked hydrogel (Lipo/CXB@Hydrogel) offers excellent self-healing, tissue adhesion, mechanical stability, and tunable degradation for post-surgical application.
• **Sustained Release & Improved Delivery:** Single in-situ implantation provides precise, prolonged CXB release (extending duration from 4 h to 2 weeks), overcoming limitations of frequent dosing and monotherapy resistance.
• **Synergistic Immuno-Metabolic Therapy:** Releases CXB to suppress inflammation and enhance CTL infiltration, while Lipo blocks tumor fatty acid uptake in OTME, synergistically amplifying CTL activation via targeting the AA/COX-2/PGE2 axis to prevent recurrence.
• **Integrated** **Wound Management:** The hydrogel simultaneously promotes skin regeneration and effectively seals reopened wounds, addressing post-surgical healing challenges.

## Graphical abstract

**Figure:**
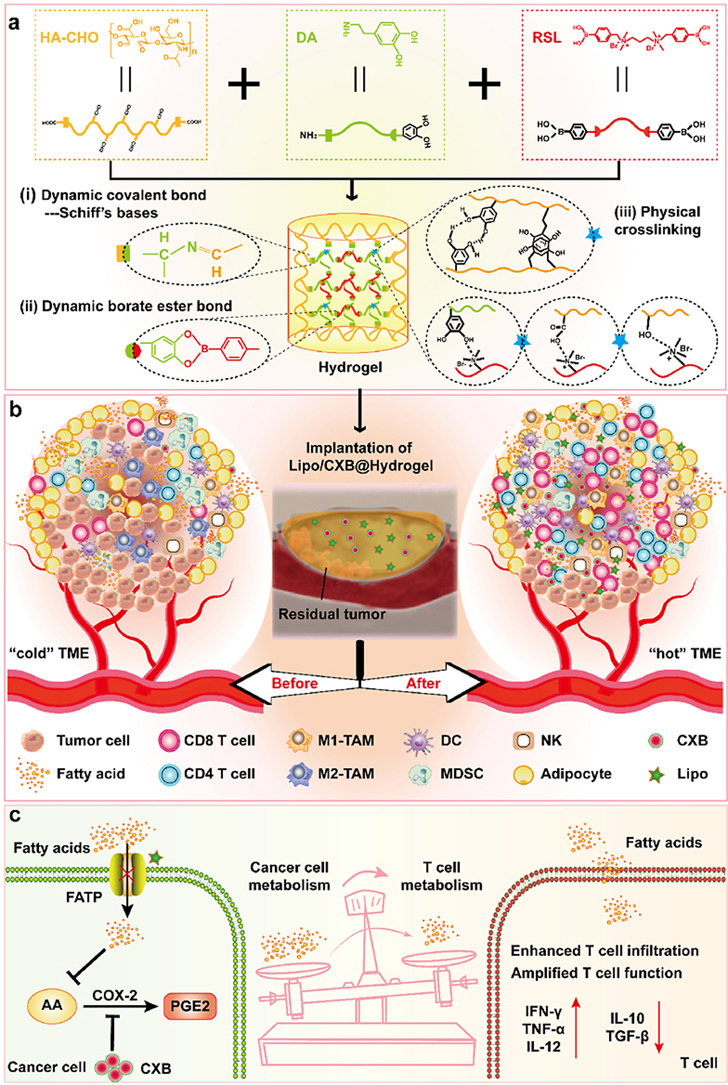

## 1 Introduction

Obesity, recognized as a high-risk factor for colorectal cancer (CRC), involves metabolic imbalance, excessive inflammation, and immune dysregulation [1,2]. Although CRC incidence has declined over the past decade, the incidence of obesity-associated CRC has progressively increased [3,4]. Surgical resection is a primary and commonly employed treatment for CRC [5,6]. However, incomplete tumor excision and deep invasive malignant components frequently cause tumor relapse or metastasis in spite of modern operative methods having undergone substantial refinements [7,8]. In addition, a long and infection-susceptible healing period of postsurgical large wounds often leads to unsatisfactory tissue repair and obviously increases the risk of complications [9,10]. Consequently, developing a therapeutic strategy that could simultaneously eradicate residual tumors and promote wound repair is essential for CRC treatment.

The rampant proliferation of cancer cells demands substantial energy, relying on diverse metabolic pathways such as carbohydrate, nucleic acid, amino acid, and lipid metabolism [11]. Previous studies proposed that tumor cells preferentially utilize glycolysis for energy production, suggesting that inhibiting glycolysis could “starve” tumors [12,13]. However, recent research reveals that obesity-driven tumors undergo extensive lipid metabolic reprogramming, with strong addiction to lipid uptake and fatty acid oxidation for energy supply [[14], [15], [16]]. Notably, the metabolic tug-of-war between cancer cells and antitumor T cells in the obese CRC microenvironment showed that cancer cells could upregulate fatty acid transport proteins (FATPs) to uptake and deprive free fatty acids, but CD8^+^ T cells could not, which results in fatty acids in the tumor microenvironment (TME) being depleted and T cells becoming dysfunctional due to a lack of this essential fuel [17]. In addition, previous studies demonstrated postsurgical TME enriched neutrophils elevated the uptake of arachidonic acid (AA) and the synthesis of prostaglandin E2 (PGE2) by overexpression of FATPs to facilitate their immunosuppressive capabilities [18,19]. These different lipid metabolic adaptations indicate that FATP overexpression represents both a critical metabolic barrier and a key vulnerability to prevent obese CRC growth and enhance anti-tumor immune effectiveness.

Concurrently, surgical wound-induced inflammatory responses promoting metastatic recurrence have been widely observed [20,21]. Obesity easily leads to a chronic metabolic inflammatory syndrome and exacerbates postoperative inflammation, playing a pivotal role in CRC recurrence, metastasis and wound infection [22]. This aberrant inflammation presents a particular challenge: it simultaneously sustains a pro-tumoral immune contexture while impeding the ordered process of tissue repair. Cyclooxygenase-2 (COX-2) and its downstream lipid metabolite prostaglandin E2 (PGE2), which serve as potent inflammatory mediators, are markedly upregulated in CRC to aggravate tumor cell survival, proliferation, and metastasis [23,24]. Furthermore, the COX-2/PGE2 signaling plays a main role in immunosuppressive TME through exacerbating the immunosuppressive activities of myeloid-derived suppressor cells (MDSCs) and tumor-associated macrophages (TAMs), polarizing them towards an M2-like, pro-tumoral phenotype, directly inhibiting cytotoxic cell function and aggravating tumor immune evasion [[25], [26], [27]]. Previous studies highlight the clinical potential of selective COX-2 inhibitor celecoxib (CXB) in CRC treatment, demonstrating anti-inflammatory, analgesic effects and enhanced antitumor immunity of CXB [28,29]. Importantly, Victoria et al. revealed that CXB could reprogram the immune tumor microenvironment by effectively boosting antitumor CTLs and interferon gamma (IFN-γ) responses [30]. However, this effect is transient, evidenced by tumor suppressor gene expression peaking within 4 h post-administration but rapidly declining to near-baseline levels within 24 h. This underscores the need for localized therapeutic platforms with superior drug-loading capacity and sustained release properties.

**Scheme 1:**
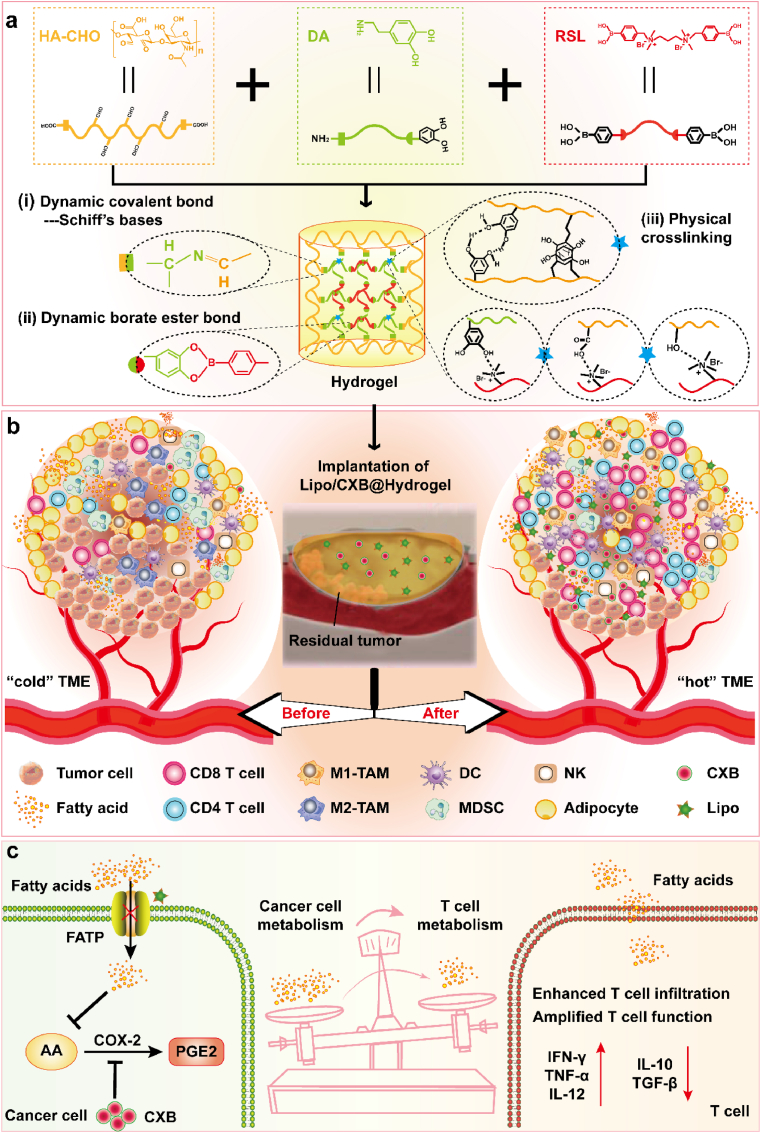
Lipo/CXB@Hydrogel treatment reshapes the obesity-associated TME to facilitate cancer postsurgical immunotherapy. (a) Schematic diagram of the formation of the multiple cross-linking hydrogel. (b) The implantation of Lipo/CXB@Hydrogel can efficiently ameliorate the immunosuppression of obese TME. (c) Lipo/CXB@Hydrogel modulates lipid metabolism and inflammation through suppressing AA/COX-2/PGE2 pathway, redirects the fatty acid energy toward T lymphocytes, thus promoting their penetrative accumulation and tumor cytotoxicity in the obese TME via immunometabolic crosstalk.

For effective obese CRC postoperative immunotherapy and wound healing, several factors must be considered when using topical treatment platforms due to colorectal anatomy and physiology. First, excellent safety and tissue compatibility without interfering with wound healing; Second, robust interfacial adhesion ensures prolonged immobilization within the surgical wound bed; Third, therapeutic payloads require controlled and sustained release kinetics to maintain therapeutic efficacy. To this end, a dual-drug depot (Lipo/CXB@Hydrogel) consists of interpenetrating polymer networks composed of oxidized hyaluronic acid (HA-CHO) and reactive oxygen species (ROS)-sensitive linker (RSL) after cross-linking with dopamine, in which co-encapsulated CXB and FATP inhibitor Lipofermata (Lipo). The hydrogel depot constructed by the multiple cross-linking network strategy has characteristics of excellent histocompatibility, strong adhesiveness and self-healing, stable mechanical strength and controllable degradation, which is an ideal choice as an in-situ drug delivery system for obese CRC postsurgical immunotherapy and wound management. The released CXB not only effectively reduces hyperinflammation in TME but also increases the intratumoral accumulation of effector T cells and builds a “hot” microenvironment conducive to the immune response. Meanwhile, the consistently released Lipo “starves” tumor cells to provide enough lipid energy for accumulated T cells to amplify antitumor immunity. A pivotal discovery of this study lies in the synergistic application of Lipo and CXB, which markedly improves immunotherapy efficacy against obese CRC by regulating lipid metabolism and inflammatory responses via suppression of the arachidonic acid (AA)/COX-2/PGE2 axis. This approach of continuously releasing the therapeutic payloads locally not only overcomes the transient therapeutic effects and long-term multiple-dose administration of CXB monotherapy but also addresses the limited effectiveness and therapeutic resistance inherent to standalone starvation therapy. In addition, in a full-thickness skin incision model, implanted Lipo/CXB@Hydrogel is capable of repeatedly closing the reopened wounds and accelerating wound healing. Collectively, the engineered multifunctional hydrogel developed herein enables local implantation at tumor resection sites to promote wound repair, achieve a reinforcing T-cell immune response for preventing postoperative recurrence and metastasis in obesity-related CRC (Scheme 1).

## 2 Results and discussion

### 2.1 Obesity accelerates postsurgical tumor growth and changes tumor immune microenvironment

**Fig. 1:**
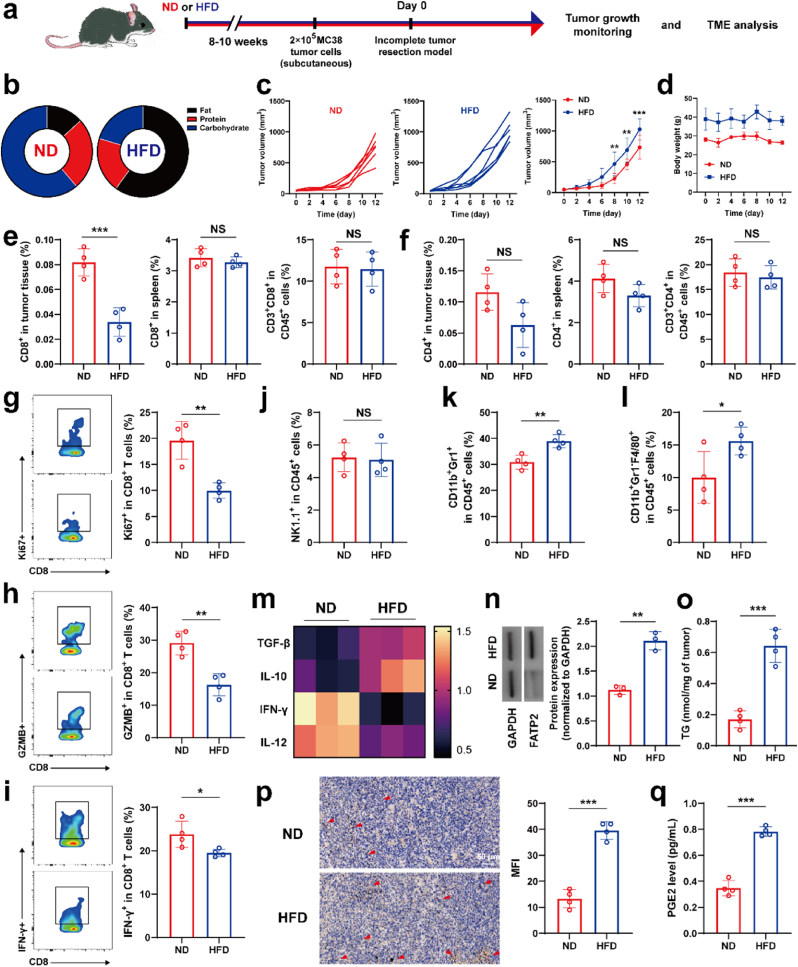
Obesity accelerates postsurgical tumor growth and exacerbates tumor immune microenvironment. (a) Schematic illustration of experimental design. (b) Comparison of fat, protein, and carbohydrate in ND and HFD diet. (c) Individual and average postoperative tumor growth curves (n = 6). (d) The average body weight of mice (n = 6). (e) Quantification of the percentage of CD8^+^ T cells and CD4^+^ T cells (f) in tumor, spleen and lymph nodes (n = 4). (g–i) Quantification of Ki67 (g), GZMB (h), and IFN-γ (i) expression on intratumoral CD8^+^ cells (n = 4). (j–l) The ratio of NK cells (j), MDSCs (k) and TAMs (l) among intratumoral CD45^+^ cells (n = 4). (m) The heatmap of cytokines within tumor from ND and HFD mice (n = 3). (n) Representative western blots and the corresponding semi-quantitative results of FATP2 within tumor from ND and HFD mice (n = 3). (o) TG contents in MC38 tumors from ND and HFD mice (n = 4). (p) Immunohistochemical staining and quantifications of COX-2 signal in HFD and ND tumors (n = 4). (q) PGE2 level in MC38 tumors from HFD and ND mice (n = 4).

Obesity is a risk factor for numerous cancers, such as colorectal, breast, liver and pancreatic cancer [31,32]. Following randomization at four weeks of age, C57BL/6 mice were assigned to receive either a normal diet (ND, 10 %-fat diet) or a high fat diet (HFD, 60 %-fat diet) to model human obesity. After 8–10 weeks of feeding, HFD mice obtained obvious increased body weight (Fig. S1a), subcutaneous, epididymal, perirenal, mesenteric, shoulder blade white fat, but decreased shoulder blade brown fat tissue (Fig. S1b and c). Meanwhile, systemic obesity-related metabolic changes were observed in HFD mice, such as hypercholesterolemia (Fig. S1d), low adiponectin (Fig. S1e), mild hyperglycemia (Fig. S1f) and slightly decreased insulin levels (Fig. S1g). To understand the effects of obesity on tumor recurrence, we first established an incomplete tumor resection model on MC38 CRC tumors at the same primary tumor sizes in ND or HFD mice (Fig. 1a and b). Not surprisingly, MC38 tumors showed more rapid tumor growth in mice with HFD feeding compared to ND (Fig. 1c), but without obvious body weight changes (Fig. 1d). Seven days after surgery, the tumors from ND and HFD mice were collected to explore how obesity influences the postsurgical tumor immune microenvironment. Compared to the ND tumor, the proportions of tumor-infiltrating CD3^+^CD8^+^ T cells in the HFD model were significantly reduced (Fig. 1e and S2), while this reduction in CD3^+^CD8^+^ T cells was not observed in the spleen or draining lymph node. Moreover, the number of CD3^+^CD4^+^ T cells in the tumors, spleen or draining lymph node was comparable in ND and HFD mice (Fig. 1f). To explore the impact of HFD on tumor-infiltrating CD3^+^CD8^+^ T cell functional competence and effector responses, we further assessed functional biomarkers associated with cytotoxic lymphocyte performance. Relative to ND controls, we found that CD3^+^CD8^+^T tumor-infiltrating lymphocytes of the HFD model expressed lower Ki67 (Fig. 1g), cytolytic molecule granzyme B (GZMB) and IFN-γ (Fig. 1h and i), implying depressed proliferation and functionality of T cells in an obese context.

Next, we extended our immune profiling of MC38 tumors beyond CD3^+^CD8^+^ T lymphocytes to examine HFD-induced alterations in additional immune compartments. While NK cell population density remained unchanged across dietary groups (Fig. 1j), we observed significant myeloid compartment remodeling characterized by elevated CD11b^+^ cell infiltration. This shift manifested through expansion of two immunosuppressive myeloid populations: CD11b^+^Gr1^+^ MDSCs (Fig. 1k) and CD11b^+^Gr1^-^F4/80^+^ tumor-associated macrophages (TAMs) (Fig. 1l). Then cytokine profiling of tumor specimens from HFD and ND was performed using ELISA-based quantification. Comparative analysis revealed a distinct immunoregulatory shift in HFD-associated tumors, characterized by depressed expression of immunostimulatory mediators (IL-12, TNF-α, and IFN-γ) coupled with enhanced production of immunosuppressive mediators (IL-10 and TGF-β) relative to ND controls (Fig. 1m). Taken together, these results provide evidence that HFD improved MC38 tumor growth and exacerbated the immunosuppressive characteristics of the TME.

Mounting evidence demonstrated that metabolic reprogramming is one of the manifesting hallmarks of most solid tumors. To adapt to the lipid-lavish microenvironment, tumor cells undergo lipid metabolism reprogramming by enhanced lipid uptake, fatty acid oxidation (FAO), and lipid storage to deprive T cells energy source and support tumor proliferation [33]. As shown in Fig. 1n and o, quantitative analyses revealed significantly elevated FATP2 protein abundance and total triglycerides (TG) accumulation in HFD-associated TME compared to ND controls. Considering the inflammation under both obesity and surgery injury could promote tumor recurrence and therapeutic resistance, the inflammation-associated indicators, including IL-1β, IL-6 and TNF-α were detected (Fig. S3), which were increased in tumors of HFD mice. In addition, COX-2 and its downstream product, PGE2, are commonly upregulated in obese tumors (Fig. 1p and q). Therefore, combining lipid metabolism and inflammation modulation could be a promising strategy to improve the obese TME immune landscape and amplify antitumor immunity.

### 2.2 Preparation and characterization of Lipo/CXB@Hydrogel

**Fig. 2:**
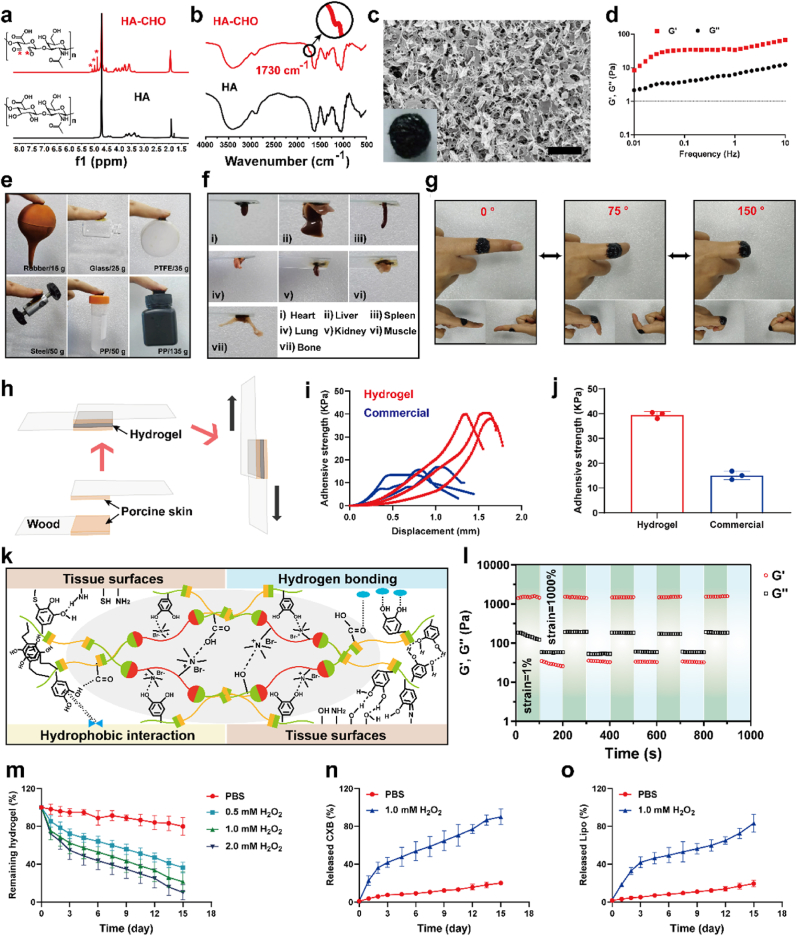
Preparation and characterization of Lipo/CXB@Hydrogel. (a) ^1^H NMR and (b) FTIR spectra showing the formation of the HA-CHO. (c) Representative SEM image of the hydrogel. Scale bar: 1 μm. (d) Angular frequency-dependent rheology of the hydrogel during the angular frequency sweep from 0.01 to 10 rad/s at 1 % strain. (e) The hydrogel was adhered to various material and (f) tissues surfaces. (g) The hydrogel was adhered to skin with variously bending degrees. (h) Schematic diagram of the lap shear test. (i) The adhesive strength curves of hydrogel and commercial fibrin sealant to porcine skins (n = 3). (j) Quantitative results of adhesive strength (n = 3). (k) The adhesion mechanism of the hydrogel. (l) Self-healing characterization of the hydrogel under rotational strain of 1 % and 1000 %, respectively. (m) Degradation profile of Lipo/CXB@Hydrogel in blank PBS and PBS with various concentrations of H_2_O_2_ (0.5, 1.0 and 2 mM) at 37 °C (n = 3). (n) The cumulative CXB and (o) Lipo release in blank PBS and PBS with H_2_O_2_ (1.0 mM) at 37 °C (n = 3).

Engineered delivery hydrogel should be a desirable strategy to locally modulate lipid metabolism and inflammation with decreased systemic toxicities [34]. Hydrogels based on HA have been widely used in various clinical tissue engineering and wound dressing because they can form macromolecular network structures to act as mechanical barriers to inhibit capillary bleeding and seepage. As previously reported, aldehydes were introduced to HA by a reaction between HA and sodium periodate [35]. Then the chemical structure of HA-CHO was examined using proton-nuclear magnetic resonance spectra (^1^H NMR) and Fourier transform infrared spectroscopy (FTIR). Initially, new proton peaks were observed at ≈4.9, 5.0, and 5.1 ppm, which were ascribed to the hemiacetalic proton formed from aldehydes and the neighboring hydroxyl groups (Fig. 2a). Analogously, FTIR analysis revealed a characteristic peak at ≈1730 cm^−1^ in HA-CHO, which corresponds to the C=O stretching vibrations of aldehydic carbonyl groups (Fig. 2b). The quaternization reaction was employed to prepare RSL and the structure of RSL was also confirmed by ^1^H NMR spectra (Fig. S4). Upon mixing HA-CHO and RSL into polydopamine (PDA) solution at room temperature, a versatile hydrogel is immediately generated because of the multiple reversible and irreversible covalent interactions and noncovalent bindings. The microscopic structure of the hydrogel was visualized using a scanning electron microscope (SEM), which exhibited a dense and irregular network with a porous structure (Fig. 2c). In addition, the values of storage moduli (G′) were consistently higher than loss moduli (G″) in the frequency sweep range from 0.01 to 10 Hz (Fig. 2d), suggesting a gel-like character and elasticity-oriented hydrogels.

Since catechol chemistry based on PDA revealed a universal strategy for preparing a hydrogel with good adhesiveness and super toughness, the adhesive performance of the hydrogel was also evaluated. The hydrogel displayed prominent adhesiveness to various substrates, including rubber, glass, Teflon (PTFE), steel and polypropylene (PP) with different weights (Fig. 2e). Meanwhile, a freshly prepared hydrogel also can adhere to diverse biological tissues, such as heart, liver, spleen, lung, kidney, muscle and bone, which is beneficial for postsurgical tumor wound dressing application (Fig. 2f). An optimal postoperative hydrogel could serve as a protective barrier onto the scarred skin, which should tightly attach to fragile surgical wounds without any detachment. As demonstrated in Fig. 2g, the hydrogel firmly adhered to the skin surface of the author's finger without any separation even with a 150° bend, indicating the high adhesion was sufficient to adapt to skin deformation. Moreover, the adhesive capability of the hydrogel was quantitatively evaluated using a lap shear test on porcine skin, a model substrate chosen for its epidermal, structural, and microbiological resemblance to human skin, as well as its collagen-rich composition, which is comparable to that of other laboratory animals like mice and rats (Fig. 2h). Compared to a commercially available fibrin glue control, the hydrogel demonstrated a superior adhesion strength of 39.5 kPa, which was 2.6 times higher than that of the fibrin glue (15.1 kPa) (Fig. 2i and j). The multifunctional adhesiveness of the hydrogel was ascribed to the adequate interface force supplied by the hydrogel polymer through hydrophobic interactions and hydrogen bonding with the substrates (Fig. 2k). Of note, disruption of the hydrated isolate layer and replacement of hydrated cations on tissue surfaces by the positive NH_4_^+^ group in the RSL of hydrogel strongly support the catechol groups of the PDA to generate both covalent binding through Schiff's base reactions or Michael addition with the nucleophiles (amines, thiol, and amide bonds) on tissue surfaces. In addition, noncovalent binding via hydrogen bonding and π–π stacking between hydrogel and tissue surfaces also synergistically favors tissue adhesion.

Owing to high dynamic reversible Schiff base reactions of HA-CHO with PDA, boronic acid ester covalent bonds of RSL with PDA, the noncovalent hydrogen bonds of HA-CHO with PDA and RSL, and π–π stacking of PDA, the hydrogel possesses excellent self-healing capacity and can vastly keep stability when subjected to external stress. After cutting the original hydrogel into three pieces, these three independent patches could be put together, contacted and recovered into an integrated one in 2 min without any stimuli (Fig. S5). After recombination, a step-strain rheological measurement was employed to quantitatively examine the self-healing property of the hydrogel with an angular frequency of 10 rad/s. As exhibited in Fig. 2l, the G′ value is always higher than G″ when under small strain (1 %), revealing a durable gel state. Nevertheless, when the strain upregulated to a larger one (1000 %), both G′ and G″ values reduced and that of G′ reduced more than G″, revealing the damage and breaking of the hydrogel. And the G′ and G″ of hydrogel could be restored when the strain force decreased to 1 % strain again. Moreover, after 4 times of step-strain rheological test, the G′ and G″ values were nearly unchanged from that of the first time, suggesting the repeatable self-healing behavior of hydrogel.

The degradation characteristics of the ROS-responsive hydrogel were investigated in phosphate buffer saline (PBS) containing gradient concentrations of hydrogen peroxide (H_2_O_2_). As shown in Fig. S6, the hydrogels remained stable in PBS (pH 7.4) or pH 6.5 buffer (mimicking the conditions of the TME) but were quickly degraded within 2 weeks in the presence of H_2_O_2_. The morphological transformation and structural disintegration of the hydrogel were observed in both elevated H_2_O_2_ levels and extended incubation durations (Fig. S7). The remaining hydrogels were weighed at the indicated time points and showed faster decomposition rates with higher H_2_O_2_ concentrations, which may be attributed to the ROS-sensitive rupture of the RSL polymer (Fig. 2m). The inflammatory cytokine release (TNF-α, IL-6) from macrophages stimulated by these ROS-degraded hydrogel fragments was further assessed. Only slightly elevated inflammatory cytokine indicated the low cytotoxicity and excellent biocompatibility of the hydrogel (Fig. S8). In addition, to examine the drug release characteristics, Lipo/CXB@Hydrogel was initially incubated in PBS with or without H_2_O_2_ (1 mM) and then the released drug concentrations were quantified by high-performance liquid chromatography (HPLC). After 15 days of immersion, Lipo exhibited cumulative release percentages of 16.4 % and 83.3 % in PBS and PBS containing H_2_O_2_ conditions, respectively (Fig. 2n). Similarly, CXB demonstrated 20.2 % and 89.3 % cumulative release under identical oxidized conditions (Fig. 2o). Collectively, these experimental findings confirm Lipo/CXB@Hydrogel's ROS-regulated sustained degradation and release behavior.

### 2.3 In-vivo antitumor efficacy of Lipo/CXB@Hydrogel

**Fig. 3:**
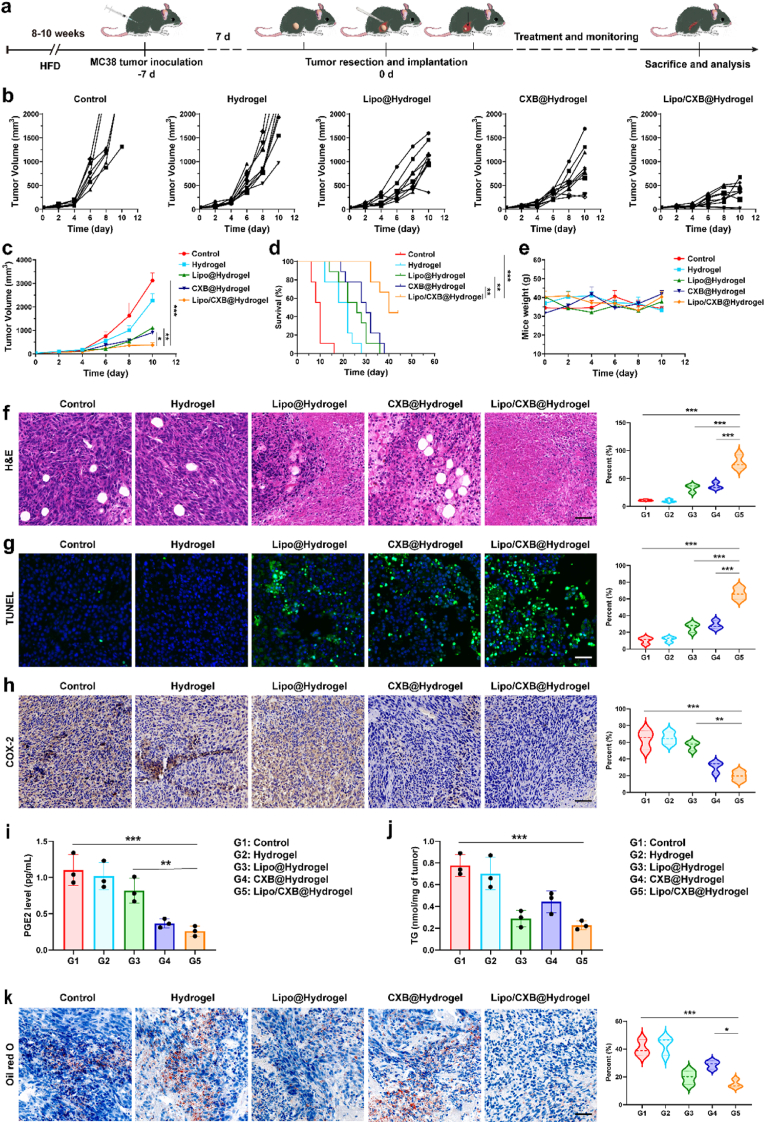
Lipo/CXB@Hydrogel suppress tumor recurrence and extends survival of HFD mice. (**a**) Schematic illustration of tumor challenge and therapeutic profile. (**b**) Individual tumor growth curves, (c) average tumor growth curves, (**d**) Kaplan-Meier survival curves and (e) average tumor weight curves for MC38 tumor–bearing HFD mice (n = 9). Mice were euthanized upon reaching a tumor volume of 2000 mm^3^, which served as the experimental endpoint. f) Representative H&E result, (g) TUNEL result and (h) COX-2 result of tumor after different treatments and the corresponding quantified percentages (n = 3). Scale bar: 50 μm. (i) The content of PGE2 and (j) TG in the tumors of mice with different treatments (n = 3). (k) Representative Oil red O staining of the tumor tissues and the quantified percentages with different treatments (n = 3). Scale bar: 50 μm.

MC38 colorectal cancer cells were subcutaneously implanted into obese murine flanks, followed by tumor resection on day 10 when lesions reached approximately 300 mm^3^ in volume. Surgical intervention involved removing 90 % of the neoplastic mass while retaining 10 % of primary tumor tissue to clinically simulate incomplete excision (Fig. 3a). It should be emphasized that solitary surgical intervention rarely achieves curative outcomes in advanced oncology cases due to inevitable tumor recurrence or metastatic dissemination. All experimental subjects receiving only tumor resection and control scaffolds exhibited significant tumor regrowth, with mortality occurring within 30 days post-operation (Fig. 3b, c and d). Therapeutic regimens employing monotherapy-loaded scaffolds (either CXB alone or Lipo exclusively) delayed tumor recurrence and extended survival marginally but failed to protect recipients beyond 40 days. Notably, the integrated combination hydrogel co-loaded with both CXB and Lipo demonstrated substantial tumor progression inhibition, revealing distinct combinatorial synergy. This dual-agent system achieved complete tumor eradication in 25 % of treated subjects, with the entire cohort maintaining full viability exceeding 30 days post-treatment. Furthermore, the absence of detectable weight loss in mice treated with Lipo/CXB@Hydrogel underscores the high biocompatibility of the 3D porous hydrogel system (Fig. 3e).

To evaluate therapeutic impacts on tumor recurrence, harvested specimens underwent histopathological analysis through H&E and TUNEL staining. Quantitative assessment revealed substantial necrotic areas in tumor sections, with Lipo@Hydrogel (32.8 %) and CXB@Hydrogel (36.3 %) demonstrating significantly elevated necrosis rates compared to controls (10.7 %) and the Hydrogel group (9.7 %) (Fig. 3f). The combinatorial treatment cohort exhibited enhanced therapeutic outcomes, particularly Lipo/CXB@Hydrogel showing prominent tumor necrosis (78.9 %). Complementary TUNEL-based apoptosis analysis (Fig. 3g) corroborated these findings, with maximal apoptotic signals observed in the combination therapy group, supporting the synergistic efficacy of dual-treatment approaches in managing obese colorectal cancer. Then, immunohistochemical and western blotting evaluation of COX-2 expression revealed marked suppression in the Lipo/CXB@Hydrogel cohort (Fig. 3h and S9). This biochemical alteration aligned with functional outcomes, as evidenced by a 4.22-fold reduction in PGE2 levels compared to controls on day 7 post-treatment in the Lipo/CXB@Hydrogel group (Fig. 3i). Subsequent quantitative triglyceride analysis of single-cell tumor suspensions confirmed these observations, showing 2.67- and 3.43-fold triglyceride reductions in Lipo@Hydrogel and Lipo/CXB@Hydrogel groups, respectively, versus controls (Fig. 3j). This decreased lipid result also demonstrates consistent trends found in Fig. 3f, wherein the least adipocyte infiltration is displayed by the group treated with Lipo/CXB@Hydrogel. Meanwhile, lipidomic profiling through ORO staining demonstrated significant lipid reduction in recurrent tumors from Lipo@Hydrogel and combination therapy groups relative to untreated controls and monotherapy cohorts (Fig. 3k). These findings substantiate that the combination of COX-2 and FATP inhibition maximized the therapeutic effect and displayed outstanding suppression of tumor regrowth.

We further explored the drug distribution in the tumor, major organs and blood after in situ implantation of Lipo/CXB@Hydrogel. The highest CXB and Lipo concentrations in tumor tissues validated that abundant drug was released and retained in the local tumor (Fig. S10), highlighting the potential of drug-loaded postoperative hydrogel for favorable drug distribution in targeted tissues. Moreover, it is also crucial to evaluate the in vivo biosafety of postsurgical in situ therapeutic interventions. Longitudinal analyses revealed that liver function parameters (alanine aminotransferase [ALT], alkaline phosphatase [ALP], aspartate aminotransferase [AST]) and renal biomarkers (blood urea nitrogen [BUN], uric acid [UA], creatinine [CRE]) displayed gradual yet stable elevation patterns across all experimental cohorts during the 10-day observation period (Fig. S11). Comparative assessment of control, Hydrogel, Lipo@Hydrogel, CXB@Hydrogel, and Lipo/CXB@Hydrogel groups showed no statistically significant intergroup variations. Furthermore, systemic metabolic alterations associated with obesity pathophysiology-including hyperglycemia, hypercholesterolemia, and circulating level modulations of resistin, leptin, adiponectin, and IL-6-maintained remarkable consistency across treatment arms (Fig. S12). These findings collectively indicate that the Lipo/CXB@Hydrogel in situ implantation demonstrates favorable biocompatibility with negligible systemic toxicity or adverse physiological impacts.

### 2.4 In-vivo antitumor immune response of Lipo/CXB@Hydrogel

**Fig. 4:**
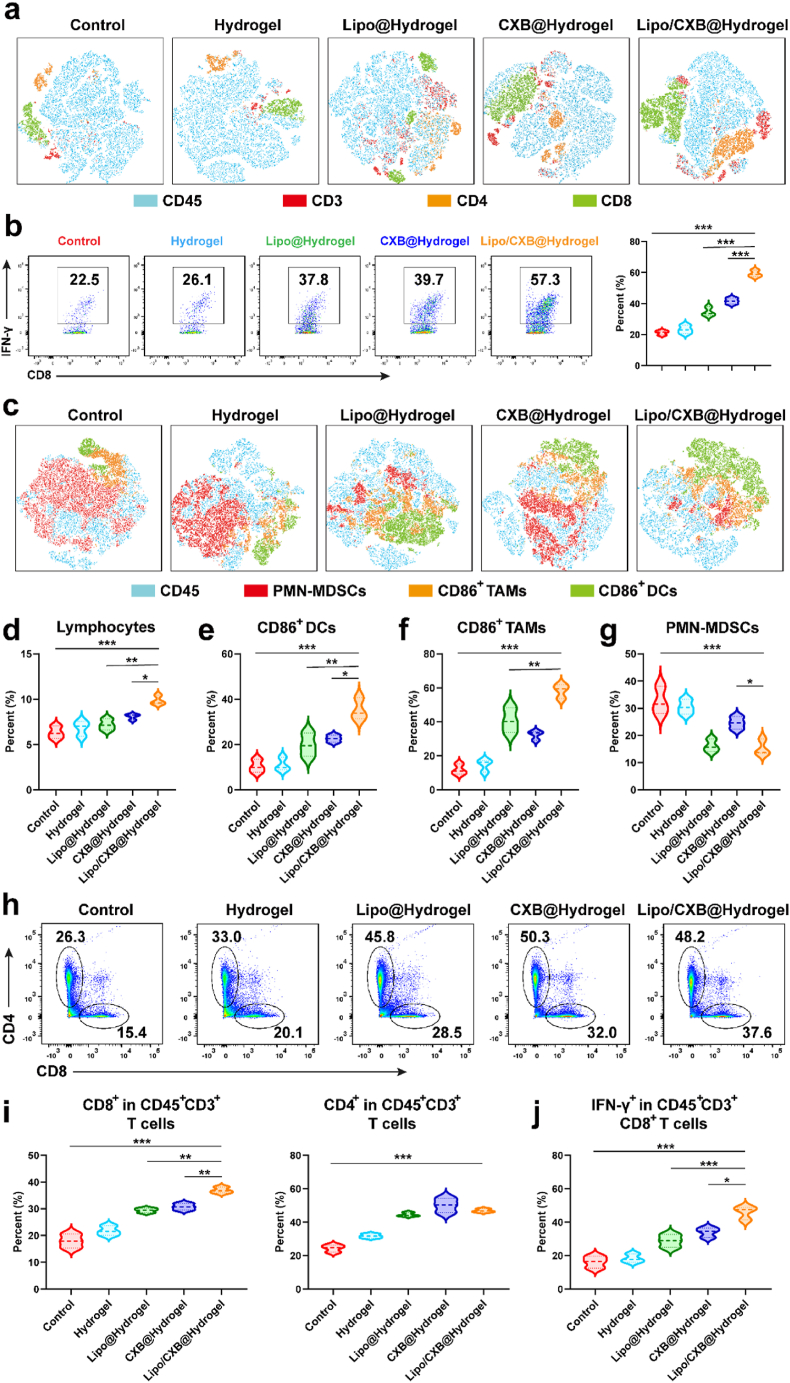
In vivo immune-activating performance of Lipo/CXB@Hydrogel. (a) t-SNE analysis of T cell subpopulations in tumor tissues after treatment. (b) Intratumoral levels of cytotoxic T cells (CD45^+^CD3^+^CD8^+^IFN-γ^+^) after treatment (n = 3). (c) t-SNE analysis of M1 TAMs, matured DCs and PMN-MDSCs in tumor tissues after treatment. (d) Percentages of lymphocytes (CD45^+^), (e) matured DCs (CD45^+^CD11c^+^CD86^+^), (f) M1 TAMs (CD45^+^CD11b^+^F4/80^+^CD86^+^) and (g)PMN-MDCSs (CD45^+^CD11b^+^Ly6G^+^Ly6C^−^) in tumors after treatments with PBS, Hydrogel, Lipo@Hydrogel, CXB@Hydrogel and Lipo/CXB@Hydrogel (n = 3). (h) Flow cytometry of CD3^+^CD8^+^ and CD3^+^CD4^+^ T cells in spleen after treatments with PBS, Hydrogel, Lipo@Hydrogel, CXB@Hydrogel and Lipo/CXB@Hydrogel. (i) Percentages of CD3^+^CD8^+^ and CD3^+^CD4^+^ T cells spleen after treatments with PBS, Hydrogel, Lipo@Hydrogel, CXB@Hydrogel and Lipo/CXB@Hydrogel (n = 3). (j) Percentages of cytotoxic T cells (CD45^+^CD3^+^CD8^+^IFN-γ^+^) in spleen after treatments (n = 3).

Seven days post-treatment, tumors were surgically removed, processed into suspensions, and labeled with fluorescence-conjugated antibodies for flow cytometry. Effector T lymphocyte infiltration is a critical determinant in achieving tumor eradication via immune activation. Analysis of intratumoral T lymphocytes revealed elevated proportions of CD8^+^ and CD4^+^ T-cell subsets within CD45^+^ leukocytes across treatment groups, with enhanced tumor suppression correlating with greater T-cell infiltration (Fig. 4a and S13). Notably, the Lipo@Hydrogel (25.1 %), CXB@Hydrogel (33.1 %), and Lipo/CXB@Hydrogel (40 %) groups exhibited significantly higher ratios of cytotoxic IFN-γ^+^CD8^+^ T lymphocytes compared to controls (Fig. 4b), suggesting that tumor-infiltrating T cells preserved robust antitumor functionality rather than becoming exhausted in the immunosuppressive microenvironment. Further flow cytometric evaluation assessed lymphocyte infiltration patterns, dendritic cell (DC) activation status, and tumor-associated macrophage (TAM) polarization. The Lipo/CXB@Hydrogel formulation demonstrated superior efficacy in enhancing intratumoral lymphocyte accumulation (Fig. 4c, d and S14a). While both Lipo@Hydrogel and CXB@Hydrogel monotherapies enhanced DC maturation, maximal maturation occurred with combination therapy (Fig. 4c–e and S14b), optimizing antigen presentation capacity for T-cell activation. Concurrently, flow cytometric analysis demonstrated that upregulated tumoral frequency of antitumor M1 macrophages (Fig. 4c–f and S15a) but downregulated frequency of protumor M2 macrophages (Fig. S15b). Moreover, we observed that Lipo/CXB@Hydrogel also increased the intratumoral abundance of NK cells (Fig. S15c), indicating a robust immune response was initiated in vivo.

Investigations into MDSCs have demonstrated that blocking FATP2 impairs the immunosuppressive capacity of polymorphonuclear MDSCs (PMN-MDSCs), thereby selectively attenuating their activity and enhancing cancer immunotherapy efficacy [18]. In this study, a marked decrease in PMN-MDSC frequency was observed in both the Lipo@Hydrogel and combination Lipo/CXB@Hydrogel groups (Fig. 4c–g and S16). Furthermore, the combined therapy significantly upregulated production of IFN-γ and interleukin (IL)-12 (Fig. S17a and b), cytokines indicative of DC activation, T lymphocyte stimulation, and M1 macrophage polarization, while concurrently suppressing immunosuppressive mediators IL-10 and TGF-β (Fig. S17c and d) secreted by immunosuppressive cells including TAMs and MDSCs. We further analyzed effector T cells in the spleen after different treatments (Fig. S18). Splenic effector T-cell profiling post-treatment revealed maximal CD8^+^ and CD4^+^ T-cell ratios in the Lipo/CXB@Hydrogel cohort, correlating with optimal antitumor immunity (Fig. 4h and i). Similar to the intratumoral trend, splenic IFN-γ^+^CD8^+^ cytotoxic T-cell populations were substantially elevated in this group (Fig. 4j), exhibiting a 2.45-fold increase over PBS controls. In addition, the abundance of memory T cell (CD3^+^CD44^+^CD62L^+^) in Lipo/CXB@Hydrogel treated mice markedly increased compared with that of PBS control (Fig. S19), revealing that Lipo/CXB@Hydrogel could elicit a durable immune memory effect for tumor regrowth prevention. Collectively, these findings demonstrate that the combined Lipo and CXB hydrogel system functions as a potent immunomodulatory platform to counteract tumor-induced immunosuppression and activate systemic immune responses against malignancies.

### 2.5 Lipo/CXB@Hydrogel reprograms tumor lipid metabolism through AA/COX-2/PGE2 axis

**Fig. 5:**
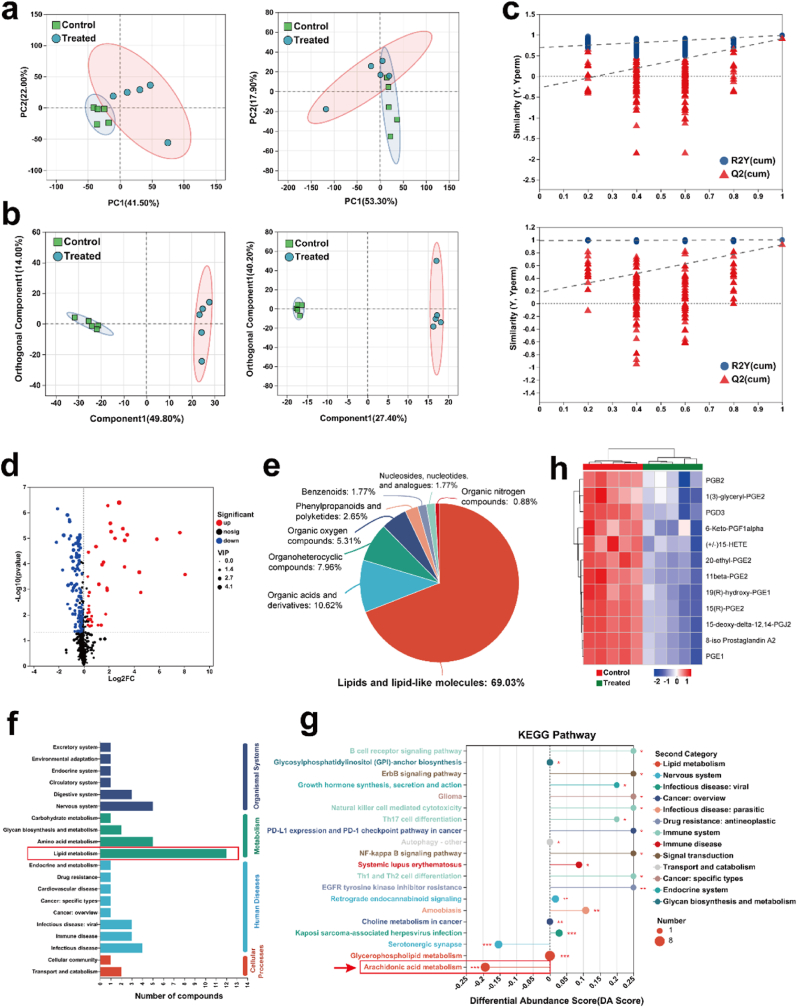
Lipo/CXB@Hydrogel reprograms tumor metabolism through AA/COX-2/PGE2 axis. (a) PCA analysis of differentially expressed metabolites in positive (left) and negative (right) ionization mode (n = 5). (b) OPLS-DA score plot of differentially expressed metabolites in positive (left) and negative (right) ionization mode (n = 5). (c) The permutation test graph of the OPLS-DA in positive (up) and negative (down) ionization mode (n = 5). (d) Volcano plot of differentially regulated expressed metabolites between untreated- and Lipo/CXB@Hydrogel-treated obese MC38 tumors (n = 5). (e) Chemical compound classification of enriched metabolic metabolites between untreated- and Lipo/CXB@Hydrogel-treated obese MC38 tumors (n = 5). (f) The top 20 metabolic functional pathways of differentially expressed metabolites between untreated- and Lipo/CXB@Hydrogel-treated obese MC38 tumors (n = 5). (g) The top 20 metabolic enriched pathways analysis of lipid metabolism between untreated- and Lipo/CXB@Hydrogel-treated obese MC38 tumors (n = 5). (h) Heatmaps of the differential arachidonic acid metabolism-related metabolites untreated- and Lipo/CXB@Hydrogel-treated tumors (n = 5).

The strong association linking obesity and CRC growth prompted an investigation on metabolic alterations in tumors after Lipo/CXB@Hydrogel treatment. To uncover potential metabolic processes in Lipo/CXB@Hydrogel-treated tumors, comprehensive metabolomic analysis (differentially expressed metabolites and enrichment analysis) was performed on tumor tissues collected at day 7. Principal component analysis (PCA) in both cationic mode and anionic mode confirmed the metabolic separation between untreated- or Lipo/CXB@Hydrogel-treated obesity-associated CRC tumors (Fig. 5a–S20 a, S20b and S20c). The results were combined with orthogonal partial least squares discriminant analysis (OPLS-DA) scores (Fig. 5b) and permutation tests (Fig. 5c) indicate that the model established in this study has high predictive ability and good stability. Based on our predefined criteria (p < 0.05, VIP >1, and |log2 (fold change)| > 1), 150 metabolites were shown to be differentially expressed (Fig. S20d), among which 40 were upregulated and 110 were downregulated (Fig. 5d). These differentiated metabolites were identified, analyzed, and their proportions were (Fig. 5e): lipids and lipid-like molecules (69.03 %), organic acids and derivatives (10.62 %), organoheterocyclic compounds (7.96 %), organic oxygen compounds (5.31 %), phenylpropanoids and polyketides (2.65 %) and benzenoids (1.77 %).

Consistent with the above results, the primary metabolic pathways identified in the treated group mainly focused on lipid metabolism pathways (Fig. 5f). The obvious changed lipid metabolism pathway suggests that tumors may reprogram their lipid metabolism after Lipo/CXB@Hydrogel treatment. We next performed Kyoto Encyclopedia of Genes and Genomes (KEGG) enrichment analysis to summarize annotated biological processes of these metabolites (Fig. 5g). It was found that there was a significantly reduced arachidonic acid (AA) metabolism after Lipo/CXB@Hydrogel treatment. In contrast with this, the abundance of antitumor immunity pathways involved in Th1 and Th2 cell differentiation, Th17 cell differentiation, natural killer cell mediated cytotoxicity and B cell receptor signaling pathways was improved in the treated group. Of note, AA is a key precursor of PGE2, which not only counteracts the anti-tumor immune effects of natural immune cells such as neutrophils, monocytes and natural killer cells, but also inhibits Th1 differentiation, B cell function and T cell activation [36,37]. The same metabolomics sequencing method was employed to analyze AA metabolism-related metabolites in tumors with Lipo/CXB@Hydrogel treatment. Analysis revealed a significant reduction in AA metabolism-related metabolites, such as PGB2, PGE2, PGE1, PGD3, PGF, and 15-HETE (Fig. 5h), suggesting that Lipo/CXB@Hydrogel reprograms tumor lipid metabolism via suppressing the AA/COX-2/PGE2 axis.

### 2.6 Transcriptomic analysis of the anticancer mechanism mediated by Lipo/CXB@Hydrogel

**Fig. 6:**
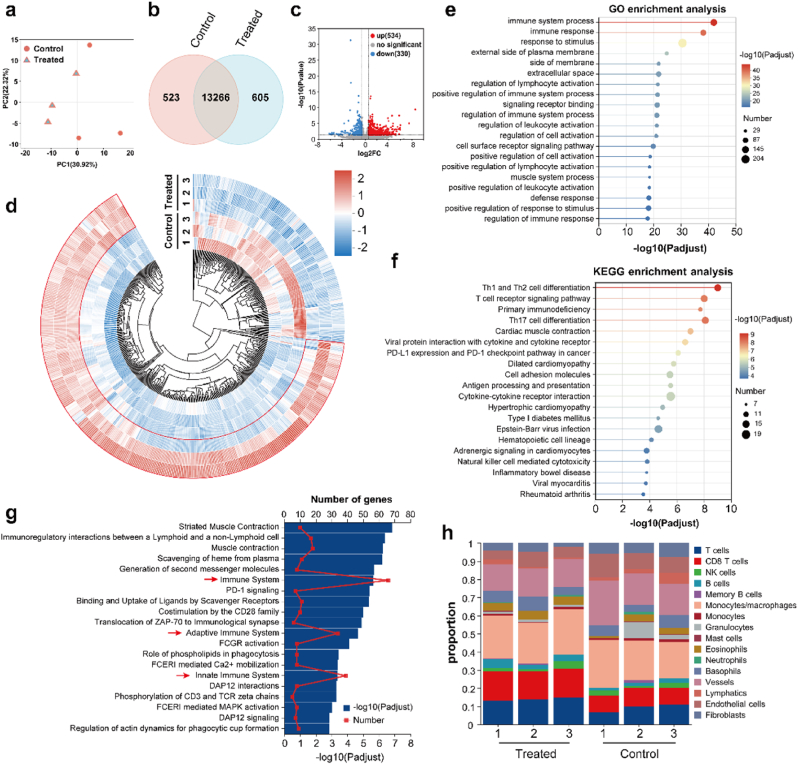
Transcriptomic analysis of the anticancer mechanism mediated by Lipo/CXB@Hydrogel. (a) PCA was carried out based on transcriptomic profiles from tumors of untreated and Lipo/CXB@Hydrogel group (n = 3). (b) Venn diagram of differentially regulated genes between untreated and Lipo/CXB@Hydrogel group (n = 3). (c) Volcano Plot of RNA-sequencing data between untreated and Lipo/CXB@Hydrogel group (n = 3). (d) Circular heatmap of differentially regulated genes between untreated and Lipo/CXB@Hydrogel group (n = 3). (e) GO enrichment analysis of top 20 terms between untreated and Lipo/CXB@Hydrogel group (n = 3). (f) TOP 20 significantly enriched KEGG pathways between untreated and Lipo/CXB@Hydrogel group (n = 3). (g) Reactome enrichment analysis between untreated and Lipo/CXB@Hydrogel group (n = 3). (h) The expression profile of tumor-infiltrating immune cells between untreated and Lipo/CXB@Hydrogel group (n = 3).

Subsequent bioinformatic characterization of miRNA transcriptome sequencing (miRNA-seq) data explored signaling pathway mechanisms connecting metabolic and antitumor immunity processes. PCA analysis revealed a significant distinction between the transcriptomes of obese CRC tumors treated with Lipo/CXB@Hydrogel and the control group (Fig. 6a). Venn diagram analysis further demonstrated differences in the total detected genes (Fig. 6b), leading to identification of 534 significantly upregulated and 330 downregulated genes (fold change ≥2, P < 0.05) in the Lipo/CXB@Hydrogel cohort versus controls (Fig. 6c). Noticeably, Lipo/CXB@Hydrogel significantly upregulated T cell activation genes (e.g., Cd28, Cd3d, Cd3e, Lck and Lat), cytotoxic T cell and NK cell functional genes (e.g., CD8a1, CD8b1, Nkg7, Tnfrsf4, Tnfsf10 and k1rk1), interferon signaling and stimulating genes (e.g., Isg15, Ifi206, Oas1a, Irgm1 and Ifi47), immune cell-recruiting genes (e.g., Cxcr6, Cxcl10, Cxcl11, Ccl4, Ccl3 and Xcl1), antigen processing and presentation genes (e.g., Tap1, Tap2, Psmb8, H2-DMa, Nlrc5 and H2-DMb1), cytokine and receptor signaling genes (e.g., Il15ra, Il18rap, Il2ra and Il12rb1), demonstrating the potent activation of antitumor immunity in vivo by Lipo/CXB@Hydrogel (Fig. 6d). Conversely, the stemness and proliferation of colorectal cancer associated genes (e.g., Lgr5, Dlk1, Fgf1, Agt and sox4), immunosuppressive genes (e.g., F13a1, Osr2, Btnl9, Vsig4 and Cma1) were obviously downregulated after treatment.

To further demonstrate the ability of Lipo/CXB@Hydrogel to stimulate the immune response pathways, Gene Ontology (GO), KEGG and Reactome enrichment analyses were conducted. Gene Ontology (GO) enrichment (Fig. 6e) and functional annotation (Fig. S21a) analyses revealed that treatment-induced alterations in differentially expressed genes predominantly implicated immune responses and metabolic pathways relative to controls. Meanwhile, analysis of differentiated genes in the KEGG enrichment analysis (Fig. 6f) and KEGG annotations analysis (Fig. S21b) revealed their close relationship with multiple immune stimulation pathways, including Th1 and Th2 cell differentiation, T cell receptor signaling pathway, Th17 cell differentiation, antigen processing and presentation, cytokine-cytokine receptor interaction and natural killer cell mediated cytotoxicity pathway. Furthermore, Reactome enrichment analysis (Fig. 6g) and Reactome annotations analysis (Fig. S21c) revealed that the changes in gene difference mainly concerned the immune system, the innate immune system and the adaptive immune system. Overall, these data validated that Lipo/CXB@Hydrogel could modulate the tumor immune microenvironment, which is strongly favorable for alleviating immunosuppression within obese TME, enhancing immune cell infiltration, and stimulating T lymphocyte activation to promote potent antitumor immune responses.

To further demonstrate the potential immunomodulation capacity of the Lipo/CXB@Hydrogel, we also determined the expression profiles of different cell types (mainly tumor-infiltrating immune cells) based on the transcriptome sequencing results of the tumor after treatment. In line with the above enrichment analysis and flow cytometric results, the data of overall cell composition (most lymphoid and myeloid cell subsets) within the tumor also demonstrated an augmentation in T cells and CD8^+^ T cells after treatment (Fig. 6h). Collectively, these combined results of transcriptomics and metabolomics indicate positive associations with T cell immune response and lipid metabolism, specifically reduced tumor AA/COX-2/PGE2 pathway, thus redirecting the fatty acid energy toward T lymphocytes.

### 2.7 Inhibition of tumor metastasis

**Fig. 7:**
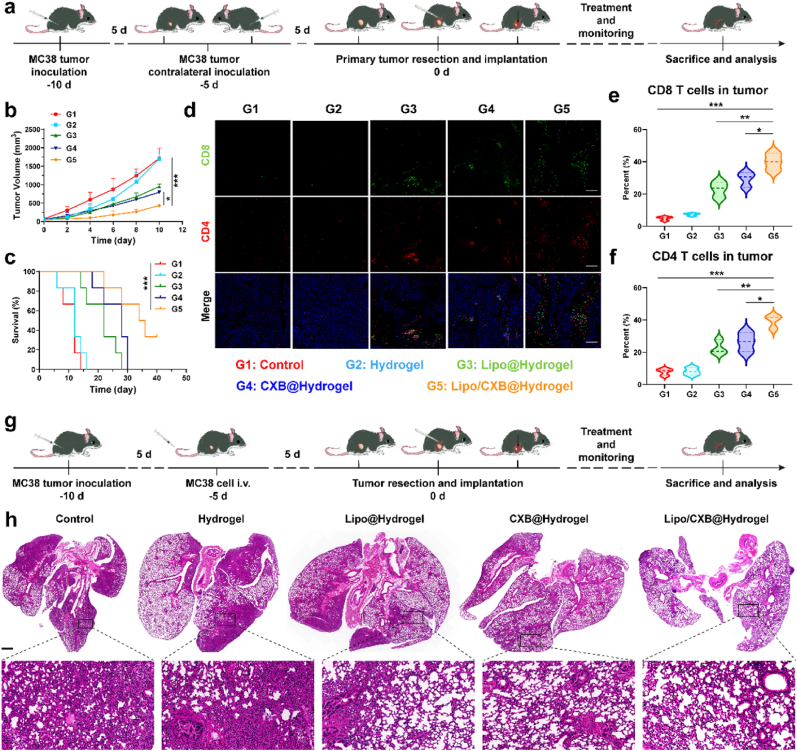
In-vivo antitumor efficacy and immune response against metastatic tumors. (a) Timeline and experimental design: (1) Dual primary/contralateral MC38 tumor-bearing murine model generation; (2) Margin-retentive tumor excision followed by in-situ hydrogel implantation, (3) Treatment, monitoring and analytical procedures. (b) Average tumor growth curves and (c) animal survival curves of the distant metastases after treatment with PBS, Hydrogel, Lipo@Hydrogel, CXB@Hydrogel, and Lipo/CXB@Hydrogel (n = 6). (d) Immunofluorescence staining of the distant tumor tissue slices. Nuclei: blue, CD4: red, CD8: green. Scale bar = 100 μm. (e) Percentages of CD8^+^ and (f) CD4^+^ T cells in distant tumors after treatment with PBS, Hydrogel, Lipo@Hydrogel, CXB@Hydrogel, and Lipo/CXB@Hydrogel (n = 3). (g) Timeline and experimental design: (1) Primary MC38 tumor-bearing and lung metastasis murine model generation; (2) Margin-retentive tumor excision followed by in-situ hydrogel implantation, (3) Treatment, monitoring and analytical procedures. (h) Representative Metastatic lung nodules in H&E-staining in mice from treated groups, including PBS, Hydrogel, Lipo@Hydrogel, CXB@Hydrogel, and Lipo/CXB@Hydrogel. Scale bar = 500 μm.

Following confirmation of the Lipo/CXB@Hydrogel system's efficacy in preventing postsurgical tumor relapse, a bilateral tumor-bearing murine model was implemented to assess its metabolic-immunotherapeutic impact on abscopal tumor suppression (Fig. 7a). Unlike comparator therapies exhibiting minimal effects on secondary lesions, the Lipo/CXB@Hydrogel cohort demonstrated pronounced inhibition of metastatic progression (Fig. 7b) accompanied by prolonged survival (Fig. 7c), thereby demonstrating robust systemic immune activation against disseminated malignancies. To investigate treatment-induced adaptive immune modulation in distal tumors, contralateral specimens were subjected to multiplex immunofluorescence analysis (Fig. 7d). Quantitative evaluation revealed enhanced T-lymphocyte infiltration in experimental groups compared to controls: Lipo@Hydrogel displayed 22.8 % CD8^+^ and 22.5 % CD4^+^ T-cell penetration, while CXB@Hydrogel achieved 28.4 % CD8^+^ and 26.5 % CD4^+^ levels, both surpassing control (5.1 % CD8^+^, 7.9 % CD4^+^) and Hydrogel-only (7.3 % CD8^+^, 8.1 % CD4^+^) cohorts. Notably, the combination therapy exhibited maximal immunocyte recruitment (40.0 % CD8^+^, 39.3 % CD4^+^), as quantified in Fig. 7e and f, confirming its capacity to orchestrate systemic antitumor T-cell responses for metastatic containment.

Building upon these findings demonstrating that this Lipo/CXB@Hydrogel can counteract both local recurrence and distal metastasis, the potential against pulmonary metastases was further systematically evaluated (Fig. 7g). Histopathological assessment of lung tissues harvested 21 days post-intervention via H&E staining (Fig. 7h) revealed a metastasis suppression gradient: control > Hydrogel > Lipo@Hydrogel > CXB@Hydrogel > Lipo/CXB@Hydrogel. Moreover, the combination group showed near-complete inhibition of nodule formation, with only residual microscopic lesions detectable. These data conclusively establish that sustained dual delivery of Lipo and CXB via hydrogel-mediated depot formation effectively maximizes the therapeutic effect through immunometabolic reprogramming of the obese tumor microenvironment.

### 2.8 In vivo wound healing

**Fig. 8:**
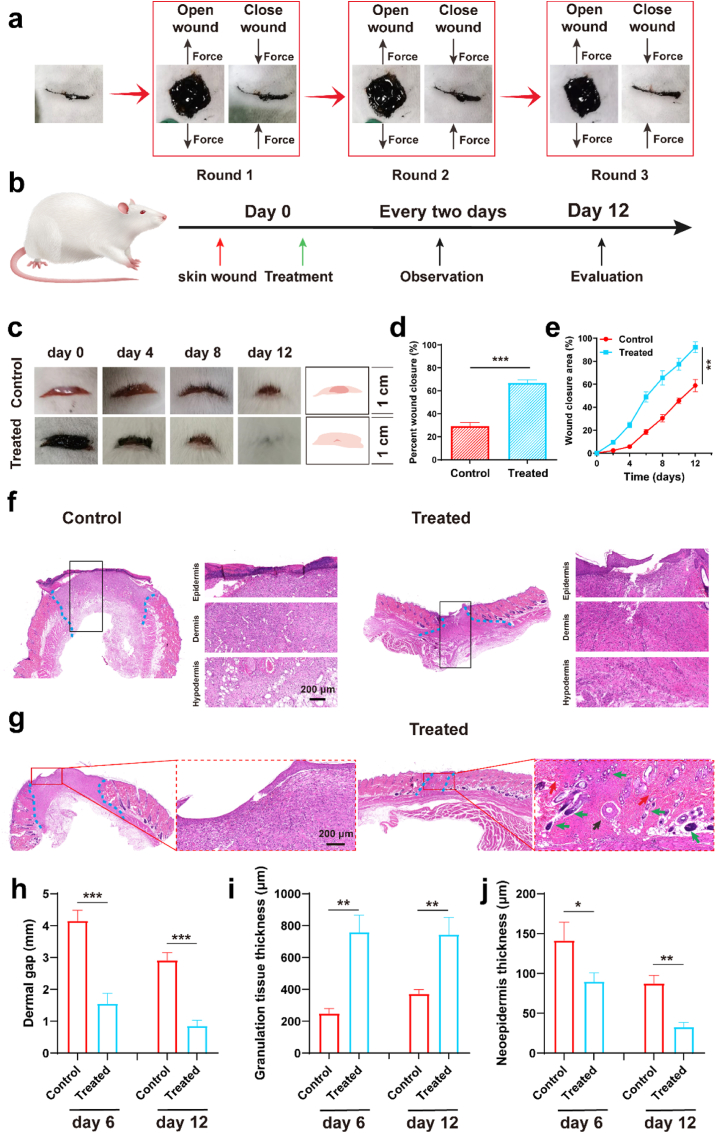
In vivo wound healing study of Lipo/CXB@Hydrogel. (a) Repeated wound healing test of Lipo/CXB@Hydrogel. (b) Schematic showing the wound treatment process. (c) The wound images of control and of Lipo/CXB@Hydrogel from day 0 to day 12. (d) Wound closure rates on day 8 for all group (n = 5). (e) Quantification of wound healing during treatment for all group (n = 5). (f) H&E staining was performed on wound regeneration samples using control and of Lipo/CXB@Hydrogel group on day 6 and (g) day 12. The blue dashed lines, black arrows, red arrows and green arrows indicate the remaining wound areas, blood vessels, collagen and hair follicles. (h) Statistics of wound length on the 6th and 12th days (n = 3). (i) Statistics of granulation tissue thickness on the 6th and 12th days (n = 3). (j) Statistics of new epidermal thickness on Day 14 (n = 3).

Surgical removal of tumors frequently necessitates the creation of incision sites. Consequently, facilitating effective wound closure following such oncological procedures is critically important. To evaluate the sealing efficacy of the Lipo/CXB@Hydrogel, a full-thickness cutaneous incision served as the experimental wound model. Initial investigations focused on assessing the hydrogel's repeat wound closure capacity. Consistent with the in vitro self-healing experiments, the hydrogel-sealed incision was subjected to deliberate mechanical separation. This induced gap was maintained under sustained tensile force for a duration of 120 s, deliberately replicating scenarios where accidental disruption of approximated wound edges might occur during the critical healing period (Fig. 8a). After removing the external force, the Lipo/CXB@Hydrogel could close the incision again. Crucially, the integrity of the re-sealed incision remained intact despite subsequent biomechanical manipulation. Furthermore, this sequential process of disruption followed by reliable re-adhesion was demonstrably repeatable for a minimum of three complete cycles. These collective observations provide compelling evidence for the hydrogel's substantial tissue-adhesive properties and robust intrinsic self-repair capability.

The full-thickness wound model was utilized to assess the therapeutic effect of the Lipo/CXB@Hydrogel (Fig. 8b). The macro images of wound changes were recorded every 4 days and displayed in Fig. 8c, and the wound closure rates were acquired by analyzing the change in wound area over time. It was discovered that the wound healing in the control group only achieved about 30.45 % closure on day 8, obviously lower than that of the Lipo/CXB@Hydrogel group (∼65.43 %), suggesting that hydrogel could accelerate the wound healing process (Fig. 8d and e). Histological analysis of the regenerated tissue was also investigated by H&E staining. By day 6, epithelial proliferation initiating from the wound edges, and the wounds in the hydrogel-treated group showed a markedly complete and stable epithelial structure (Fig. 8f). On day 12, full epithelialization had occurred in every group, and the Lipo/CXB@Hydrogel group displayed a basic structure that was similar to the original skin, which was full with newly formed blood vessels (black arrows), collagen (red arrows) and hair follicles (green arrows) (Fig. 8g). Then three critical healing parameters: wound length (Fig. 8h), granulation tissue thickness (Fig. 8i), and neoepidermis thickness (Fig. 8j) was evaluated using H&E-stained sections. The most favorable outcomes were observed in the Lipo/CXB@Hydrogel group, which displayed the shortest wound length and the greatest granulation tissue thickness. Notably, the neoepithelium in these animals was also thinner and more comparable to mature epidermis thickness by day 12. In addition, there was notable upregulation of the secretion of anti-inflammatory-related IL-10 and TGF-β after Lipo/CXB@Hydrogel treatment (Fig. S22). These findings demonstrate that Lipo/CXB@Hydrogel accelerates tissue repair and neogenesis, exhibiting considerable promise as a versatile therapeutic platform for post-resection wound management.

## 3 Conclusion

In this study, a multifunctional hydrogel composed of dopamine-crosslinked HA-CHO and RSL was developed to suppress postsurgical tumor recurrence while promoting wound healing. The unique multi-dynamic-bond crosslinked structure of the hydrogel based on dynamic covalent bonds, borate ester bonds and various physical bonds endows the hydrogel with excellent self-healing, strong tissue adhesiveness, stable mechanical properties, and tunable degradation. The sustained release of CXB from the hydrogel suppresses tumor-associated hyperinflammation through the COX-2/PGE2 axis, recruits effector T cells and transforms the immunosuppressive TME into an immunologically active niche. Meanwhile, Lipo deprives lipid energy in tumor cells by inhibiting fatty acid uptake, redirecting metabolic resources to support CTL proliferation and function. The hydrogel-based combination immunotherapy targets the AA/COX-2/PGE2 axis reprogramming inflammatory and lipid metabolism in obese TME, significantly upregulating T cell activation, cytotoxic T cell function, and stimulating signals. In addition, this in situ hydrogel addresses the transient efficacy of CXB monotherapy and enhances the limited antitumor effects of metabolic starvation strategies. In preclinical models of postsurgical obese CRC, the sustained co-delivery system significantly prolonged CTL-mediated tumor cytotoxicity, reducing recurrence and metastasis. The suppression of residual tumor removes the continuous inflammatory stimulus, thereby allowing the wound microenvironment to transition naturally towards a healing phase. Notably, the hydrogel matrix provides crucial passive support for this natural healing process through its excellent adhesion, self-healing capacity, and provision of a 3D physical structure for epithelial and tissue regeneration, without actively releasing potent pharmacological signals that might interfere with the essential anti-tumor immunity. We posit that by resolving the root cause of the conflict (the residual tumor) and supporting the subsequent natural healing process, our Lipo/CXB@Hydrogel system effectively circumvents the need for direct and contradictory manipulation of macrophage phenotypes, leading to the observed synergistic suppression of tumor recurrence and promotion of wound healing. Consequently, this hydrogel demonstrates considerable promise as a versatile therapeutic platform for obesity-associated CRC treatment through T cell antitumor immunity and wound healing promotion.

## CRediT authorship contribution statement

**Hongjuan Zhao:** Writing – original draft, Investigation, Funding acquisition, Conceptualization. **Di Meng:** Project administration, Investigation. **Yajing Wang:** Software, Project administration. **Yinke Wang:** Validation, Software. **Qing Li:** Visualization, Validation. **Yuxin Guo:** Project administration, Methodology. **Qingling Song:** Writing – review & editing, Funding acquisition. **Lei Wang:** Writing – review & editing, Funding acquisition, Conceptualization.

## Data and materials availability

All data associated with this study are present in the paper or the Supplementary Materials.

## Ethics approval and consent to participate

All the work performed on animals was following the Guidelines for Care and Use of Laboratory Animals of Zhengzhou University, and the experiments were approved by the Animal Ethics Committee of Zhengzhou University. The approval number for the study is [yxyllsc20230112].

## Declaration of competing interest

All other authors declare that they have no other competing interests.

## Supporting Information

Materials and Methods.

Fig. S1—Fig. S22 multiple supplementary figures.
