# Supplementary material for: A multifunctional hydrogel for obesity-associated tumor immunotherapy and postsurgical wound healing promotion
Source: Bioact Mater. 2025 Nov 26;57:710–25. doi: 10.1016/j.bioactmat.2025.11.027 (PMC12702186; doi:10.1016/j.bioactmat.2025.11.027)
Supplement: Multimedia component 1 [file mmc1.docx]

A Multifunctional Hydrogel for Obesity-Associated Tumor Immunotherapy and Postsurgical Wound Healing Promotion

1. **Materials**

N-hydroxysuccinimide (NHS), sodium periodate, N, N, N′, N′-tetramethyl-1,3-propanediamine, dopamine hydrochloride (DA), H_2_O_2_ (30 wt% in H_2_O), 1-ethyl-3-(3-dimethylaminopropyl)-carbodiimide (EDC) were obtained from Sigma-Aldrich (St. Louis, MO, USA). HA (MW = 200 kDa) was obtained from Xi’an Maoda Biological Technology Co.,Ltd. Ethylene glycol, dimethylformamide, 4-(bromomethyl) phenylboronic acid and tetrahydrofuran were obtained from Macklin reagent. Antibodies against cell surface markers for flow cytometry analysis were purchased from BD. Mouse uncoated enzyme-linked immunosorbent (ELISA) kits (IL-12, TNF-α, IFN-γ, IL-1β, IL-6, TGF-β and IL-10) were purchased from Thermo Fisher Scientific Co., Ltd.

1. **Preparation and characterization of HA-CHO**

The previously reported approach with slight modification was used to synthesize HA-CHO. First, 40 mg of HA (MW = 200 kDa) was dissolved in H_2_O (10 mL) and added 40 mg of sodium periodate (2 mL) aqueous solution dropwise, and the mixture was stirred for 12 h in the dark. After fully stirring, 50 µL of ethylene glycol was added to the above solution for 60 min to deactivate excess sodium periodate. Then the solution was dialyzed (MW = 10 kDa) against ultrapure water for 48 h, frozen ( -20 ℃) for 12 h and lyophilized at -80 °C to obtain HA-CHO. The aldehyde functional groups and oxidization of HA was identified by an ^1^H-NMR (AVANCE III 400 MHz, Bruker) and FTIR spectrometer (Nicolet 6700, Thermo Scientific).

1. Synthesis and characterization of RSL

The synthesis of RSL was according to our previously reported method with slight alteration. Briefly, dimethylformamide (100 mL), N, N, N′, N′-tetramethyl-1,3-propanediamine (1.0 g) and 4-(bromomethyl) phenylboronic acid (5.0 g) were mixed together and stirred overnight (60 °C). Then added the above mixture into tetrahydrofuran (1000 mL) and leave to set for at least 6 h. Then filtered the product, washed with tetrahydrofuran, and dried under vacuum to obtain RSL. Finally, the ^1^H-NMR was employed to confirm the successful synthesis of RSL.

1. **Fabrication and characterization of Lipo/CXB@Hydrogel**

HA-CHO (5.0 wt%) and DA (5.0 wt%) were dissolved in tris buffer (1 mL, pH 8.5) and then mixed together with constant stirring for 24 h to get the homogeneous mixtures. Then added 100 µL of RSL (1.25 wt%) to the above mixtures to form hydrogel instantly. The hydrogel was freeze-dried to examine the morphology by a field emission scanning electron microscope (QUTAN FEG 250, FEI). Lipo or CXB solution and RSL solution were added into mixture simultaneously and to form Lipo@Hydrogel, CXB@Hydrogel or Lipo/CXB@Hydrogel.

1. **Adhesive, rheological and self-healing performance**

Adhesive experiment: To test the adhesive ability of the hydrogel, the hydrogel (10 mm diameter and 1 mm height) was adhered with finger, and another side of the hydrogel was used to glue different hydrophilic and hydrophobic materials with various weight. To assess the adhesiveness to biological tissue, we paste glass on one side and different tissues on the other side of the hydrogel. The adhesive capacity of the hydrogel was also assessed through lap shear testing conducted on a universal testing system (AGS-X, Shimadzu, Japan) when a commercially obtained fibrin glue employed as the control. Specifically, 100 μL of hydrogel was applied between two sections of porcine skin (adhesive substrate), which secured onto wooden plates using ethyl α-cyanoacrylate. Then incubated at 37 °C for 2 hours under a 200 g load, and determined the adhesive performance of the hydrogel at a displacement rate of 1 mm/min.

Rheological test：The rheological experiment was performed on a strain-controlled rheometer (MCR302, Anton Paar, Austria). The hydrogel (20 mm diameter and 1 mm height) was placed between parallel plates, the storage modulus (G′) and loss modulus (G″) were studied by the frequency sweep tests (ω = 0.01–100 rad/s) with the strain of 1%.

Macroscopic self-healing test: The macroscopic assessment was carried out to investigate the self-healing properties of the hydrogel. Briefly, the hydrogel (10 mm in diameter and 5 mm in thickness) was divided into thirds, and then put them together one by one at room temperature. The hydrogel pieces were reassembled and underwent spontaneous self-repair without any intervention. The digital camera was used to capture the image of the hydrogel disk after 5 min.

Quantitative self-healing experiment: The hydrogel (20 mm in diameter and 1 mm in thickness) was employed to conduct alternate strain sweep test at a fixed angular frequency of 1 rad/s. The test using the strain amplitude sweep method (γ from 1% to 1000%) with 100 s time interval of each strain test. Five cycles were performed in total.

1. **In vitro** **degradation behavior of hydrogels**

The Lipo/CXB@Hydrogel (100 mg) were immersed in PBS (0.01 M, pH 7.4 or 6.5), PBS (pH 7.4) with different concentrations of H_2_O_2_ (0, 0.5, 1.0 and 2.0 mM) at room temperature. The degradation behavior of hydrogels was evaluated by photography every 3 days. Meanwhile, the residual hydrogels were collected, lyophilized and weighed to assess the degradation ability of hydrogels.

1. **ROS-responsive drug release**

The Lipo/CXB@Hydrogel (100 mg) were immersed in PBS or PBS contained H_2_O_2_ (1.0 mM) in a 37 °C incubator. At predetermined time points, supernatant (0.2 mL) was extracted for analysis and replaced with same volume of fresh media. The collected medium was centrifuged and the amount of released Lipo and CXB were analyzed by HPLC.

**8. Raising procedure of ND and HFD mice**

Female C57BL/6 mice (4 weeks old) were randomly divided into two dietary groups: controls received standard chow (ND, #5053 PicoLab Rodent Diet 20; Lab Diet) while experimental subjects were fed a high-fat regimen (HFD, #12492; 60% kcal from fat, Research Diets Inc.) for 8-10 weeks. Body mass was monitored weekly throughout the study period. All experimental protocols complied with ethical guidelines approved by Zhengzhou University's Institutional Animal Care and Use Committee (IACUC).

**9. Construction of incomplete resection tumor model**

Cultured MC38 cells were expanded in vitro and subcutaneously injected (100 μL, 2×10⁶ cells/mL) into the left flank of diet-conditioned (ND/HFD) female mice. Tumor growth progression was tracked until reaching a mean volume of ~300 mm³ (day 7 post-injection). To model clinical recurrence scenarios, 90% of established tumors were surgically resected, maintaining residual lesions to simulate incomplete tumor removal. This partial resection protocol generated a colorectal cancer recurrence model for subsequent experimental phases.

**10. Analysis of the postsurgical tumor immune microenvironment in ND and HFD mice**

MC38-bearing mice from dietary cohorts (ND/HFD) were humanely sacrificed at day 7 postsurgical for tumor immune microenvironment characterization. Excised tumors underwent mechanical dissociation in cold staining buffer to generate single-cell suspensions. To prevent nonspecific Fc receptor-mediated interactions, cell suspensions were pre-incubated with Fc-blocking anti-CD16/32 antibodies prior to subsequent immunophenotyping procedures. The single cell suspensions were stained with anti-CD45-APC-CY7, anti-CD3e-BV510, anti-CD4-PE, and anti-CD8a-PerCP-CY5.5 to analyze CD3+CD4+ T cells and CD3+CD8+T cells. The single cell suspensions were stained with anti-CD45-APC-CY7, anti-CD3e-BV510, anti-CD8a-PerCP-CY5.5 and anti-Ki67-APC (or anti-IFN-γ-BV421, or anti-GZMB-APC) for Ki67+CD8+ T cells (or IFN-γ+CD8+T cells, or GZMB+CD8+ T cells) detection. For NK cells detection, the single cell suspensions were stained with anti-CD45- APC-CY7, anti-NK1.1-PE. The single cell suspensions were stained with anti-CD45-APC-CY7, anti-Gr-1-PE-CY7, and anti-CD11b-AF488 to measure MDSCs. The single cell suspensions were stained with anti-CD45-PerCP, anti-CD11b-AF488, anti-Gr-1-PE-CY7, and anti-F4/80-BV421 to analyze tumor associated macrophages (TAMs). BD FCAS Celesta flow cytometer was utilized to collect 3.0×10^5^ cells in ungated channel of each sample, and then analyzed by FlowJo software. In addition, the tumors were cut up into single cell suspension and centrifuged (1500 r/min), the supernatant was retained and used to evaluate the level of cytokines (TGF-β, IL-10, IFN-γ, IL-12, and PGE-2) by ELISA kit, and the cell pellets were collected for western blotting assay with mouse antibody FATP2 (CST) and TG content determination by triglyceride detection kit (Beyotime). The mouse antibody COX-2 (Abcam) was immunostained with tumor tissues following the manufacturer's instructions for COX-2 evaluation.

**11.** **In vivo drug distribution**

Following local treatment with Lipo/CXB@Hydrogel (Lipo: 1.0 mg/kg, CXB: 6.0 mg/kg), tumor tissues, major organs (heart, liver, spleen, lung, kidney), and blood samples were collected at predetermined time points. The concentrations of Lipo and CXB after complete hydrolysis were detected through HPLC.

**12. In vivo anti-recurrence performance of Lipo/CXB@Hydrogel**

To assess the anti-recurrence efficacy of Lipo/CXB@Hydrogel in vivo, a subcutaneous incomplete resection model was established according to the method in part 9 and randomly divided mice into five groups (*n* = 9) immediately: (a) surgical control; (b) Hydrogel implantation; (c) Lipo@Hydrogel implantation; (d) CXB@Hydrogel implantation; (e) Lipo/CXB@Hydrogel implantation. Therapeutic monitoring included daily biometric tracking (body mass) and neoplastic growth quantification using the ellipsoid formula: Volume = (length × width²)/2. Ethical endpoints were implemented for subjects exceeding 1500 mm³ tumor burden, with mortality events incorporated into survival rate calculations.

**13. Histological analysis**

On day 7 after implantation, the collected tumors were fixed with 4 % paraformaldehyde, cut into slices, stained with H&E and immunofluorescence stained with mouse antibody TUNEL (KeyGEN BioTECH) for tumor apoptosis detection, stained with mouse antibody COX-2 (Abcam) following the manufacturer's instructions for COX-2 immunohistochemical and western blotting, Oil Red O staining (Wanleibio) for lipid assay, immunofluorescence stained with CD206 and NK for M2-TAMs and NK cells analysis.

**14. Flow cytometry analysis**

On day 7 after implantation, the recurring tumors and spleens were extracted and homogenized in cold staining buffer to form single cell suspensions for flow cytometry analysis. To prevent nonspecific Fc receptor-mediated interactions, cell suspensions were pre-incubated with Fc-blocking anti-CD16/32 antibodies prior to subsequent immunophenotyping procedures. The single cell suspensions were stained with anti-CD45-APC-CY7, anti-CD3e-BV510, anti-CD4-PE, and anti-CD8a-PerCP-CY5.5 to analyze CD3+CD4+ T cells and CD3+CD8+T cells. The single cell suspensions were stained with anti-CD45-APC-CY7, anti-CD3e-BV510, anti-CD8a-PerCP-CY5.5 and anti-IFN-γ-BV421 or anti- PE-CD44, anti- APC-CD62L for IFN-γ+CD8+T or memory T cells detection. The single cell suspensions were stained with anti-CD45-PerCP, anti-CD11c-BV605, and anti-CD86-PerCP-CY5.5 to analyze DCs. The single cell suspensions were stained with anti-CD45-APC-CY7, anti-Gr-1-BV421, and anti-CD11b-AF488 to measure MDSCs. The single cell suspensions were stained with anti-CD45-APC-CY7, anti-CD11b-AF488, anti-Gr-1-PE-CY7, anti-F4/80-BV421, and anti-CD86-PerCP-CY5.5 to analyze TAMs. BD FCAS Celesta flow cytometer was utilized to collect 3.0×10^5^ cells in ungated channel of each sample, and then analyzed by FlowJo software.

**15. Cytokine detection**

On day 7 after implantation, the tumors were extracted and dissociated into single cell suspension and centrifuged (1500 r/min), the supernatant was retained and used to evaluate the level of cytokines (TGF-β, IL-10, IFN-γ, IL-12, and PGE-2) by ELISA kit, and the cell pellets were collected for western blotting assay with TG content determination by triglyceride detection kit (Beyotime) according to the manufacturer’s instructions.

**16. Treatment safety evaluation**

To verify the biocompatibility of implantable materials, serum samples were collected through centrifugation (1,000 ×g, 20 min, 4°C) and analyzed using a Hitachi 3100 automated biochemical analyzer. This standardized protocol enabled comprehensive assessment of potential systemic toxicity through serum biomarker profiling. Meanwhile, blood glucose levels were assessed via tail vein sampling using a Contour® glucometer. For comprehensive hormonal/metabolic profiling (insulin, cholesterol, adiponectin, resistin, leptin, IL-6), terminal cardiac blood draws were performed into EDTA-anticoagulated tubes. Following centrifugation (1,500 ×g, 15 min, 4°C), plasma supernatant was aliquoted and stored at −80°C pending batch analysis via standardized immunoassays.

**17. Transcriptomics**

Tumors harvested at day 7 post-implantation (n = 3) were dissected into sterile enzyme-free microtubes for comparative transcriptomic analysis. Immune modulation in Lipo/CXB@Hydrogel-treated versus control cohorts was evaluated through mRNA sequencing. Total RNA isolation followed the standard TRIzol® protocol, with quality verification performed using dual-platform quantification: Agilent 5300 Bioanalyzer for integrity assessment and NanoDrop ND-2000 for purity/concentration measurements. Subsequent RNA processing (purification, cDNA synthesis, library preparation) and Illumina-based sequencing were conducted by Majorbio Bio-pharm Biotechnology (Shanghai, China) under standardized commercial workflows.

**18. Metabolomics**

Tumors harvested at day 7 post-implantation (n = 5) were dissected into sterile enzyme-free microtubes for comparative metabolomic analysis. The quantitative changes of tumor metabolic compounds in Lipo/CXB@Hydrogel-treated versus control cohorts was evaluated for metabolomics by Majorbio Bio-pharm Biotechnology (Shanghai, China) under standardized commercial workflows.

**19. In vivo distant metastasis inhibition of Lipo/CXB@Hydrogel**

A primary tumor was established through subcutaneous administration of 100 μL MC38 cell suspension (1×10⁶ cells/mL PBS) in the right flank. Five days post-initial injection, a secondary metastatic challenge was created via contralateral injection of identical cellular density. This sequential dual-tumor paradigm enabled simultaneous evaluation of therapeutic efficacy against both primary lesions and simulated distant metastases. Subsequently, a subcutaneous incomplete resection model was established on the primary tumor according to the method in part 9 and randomly divided mice into five groups (*n* = 9) immediately: (a) surgical control; (b) Hydrogel implantation; (c) Lipo@Hydrogel implantation; (d) CXB@Hydrogel implantation; (e) Lipo/CXB@Hydrogel implantation. Therapeutic monitoring included daily biometric tracking (body mass) and neoplastic growth quantification using the ellipsoid formula: Volume = (length × width²)/2. On day 10 after treatment, the collected tumors were fixed with 4 % paraformaldehyde, cut into slices, and immunofluorescence stained with mouse antibodies CD4 and CD8 for tumor T cell infiltration detection. Ethical endpoints were implemented for subjects exceeding 1500 mm³ tumor burden, with mortality events incorporated into survival rate calculations.

**20. In vivo lung metastasis inhibition of Lipo/CXB@Hydrogel**

A primary tumor was established through subcutaneous administration of 100 μL MC38 cell suspension (1×10⁶ cells/mL PBS) in the right flank. Five days post-initial injection, a lung metastatic challenge was created via intravenous injection of identical cellular density. Subsequently, a subcutaneous incomplete resection model was established on the primary tumor according to the method in part 9 and randomly divided mice into five groups (*n* = 9) immediately: (a) surgical control; (b) Hydrogel implantation; (c) Lipo@Hydrogel implantation; (d) CXB@Hydrogel implantation; (e) Lipo/CXB@Hydrogel implantation. On day 21 after treatment, the collected lung tissues were fixed with 4 % paraformaldehyde, cut into slices, stained with H&E for histological evaluation.

**21. In vivo wound healing study**

The *in vivo* wound healing investigation was carried out in rat model after shaving the dorsal hairs. Then 10 mm diameter full-thickness wound model was performed on the back of each rat, and randomly divided all rat into two groups (n = 5): Control group, and Lipo/CXB@Hydrogel group. Wound surface observation was applied to evaluate the regeneration procedure of wounds every 4 days, and the wound area was quantified using Image J. Cytokine profiling (TGF-β, IL-10) during healing phases was evaluated using ELISA assay.

**22. Statistical analysis**

All analyses utilized GraphPad Prism (v10.0) for hypothesis testing. All the data were expressed as mean value ± standard deviation (SD), with error bars indicating dispersion measures. Biological replicates (n≥3) ensured statistical robustness across experimental conditions. Between-group comparisons (single nominal variable) employed unpaired two-tailed t-tests. Multivariate analyses incorporated one-way/two-way ANOVA with Tukey's post hoc testing for multi-group comparisons. Survival outcomes underwent log-rank (Mantel-Cox) analysis. Significance thresholds were designated as: *P<0.05, **P<0.01, ***P<0.001.


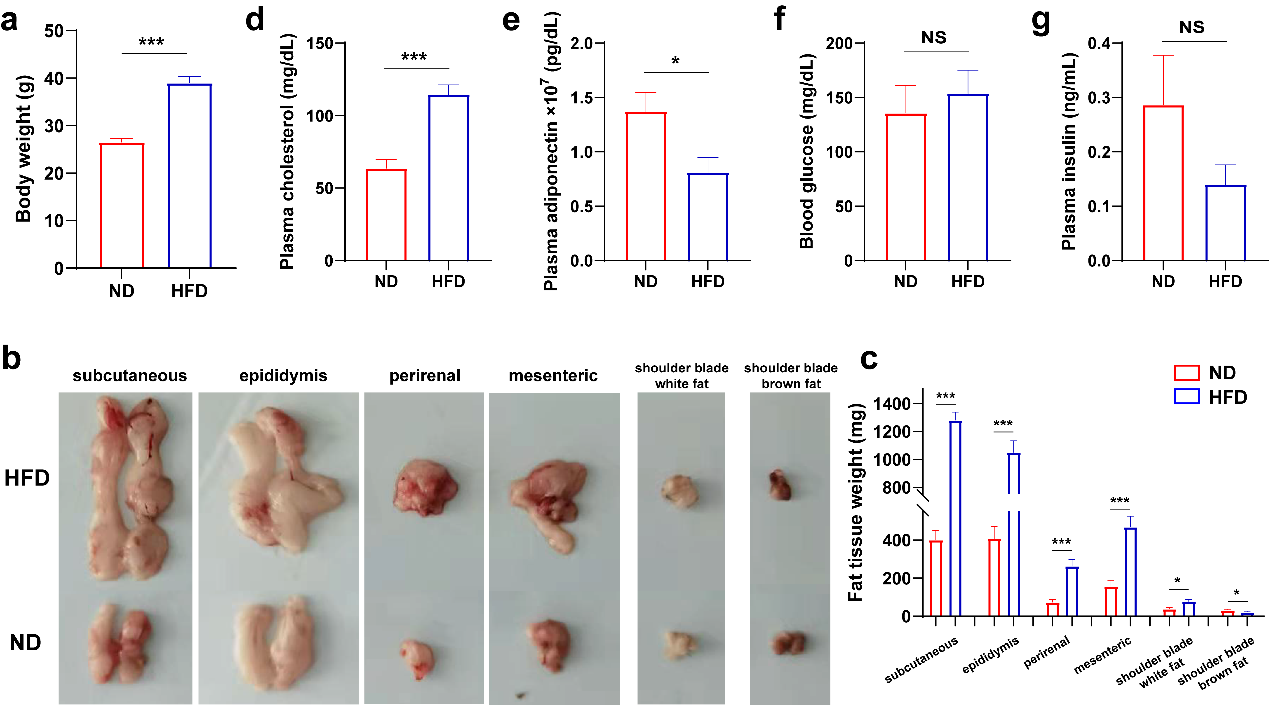


Figure S1. Effects of 8-10 weeks of dietary intervention with a ND or HFD on the C57BL/6 mice: (a) body weights (n = 5), (b) adipose tissue images, (c) fat tissue weights (n = 3), (d) plasma cholesterol (n = 3), (e) plasma adiponectin (n = 3), (f) blood glucose (n = 3), and (g) plasma insulin (n = 3).


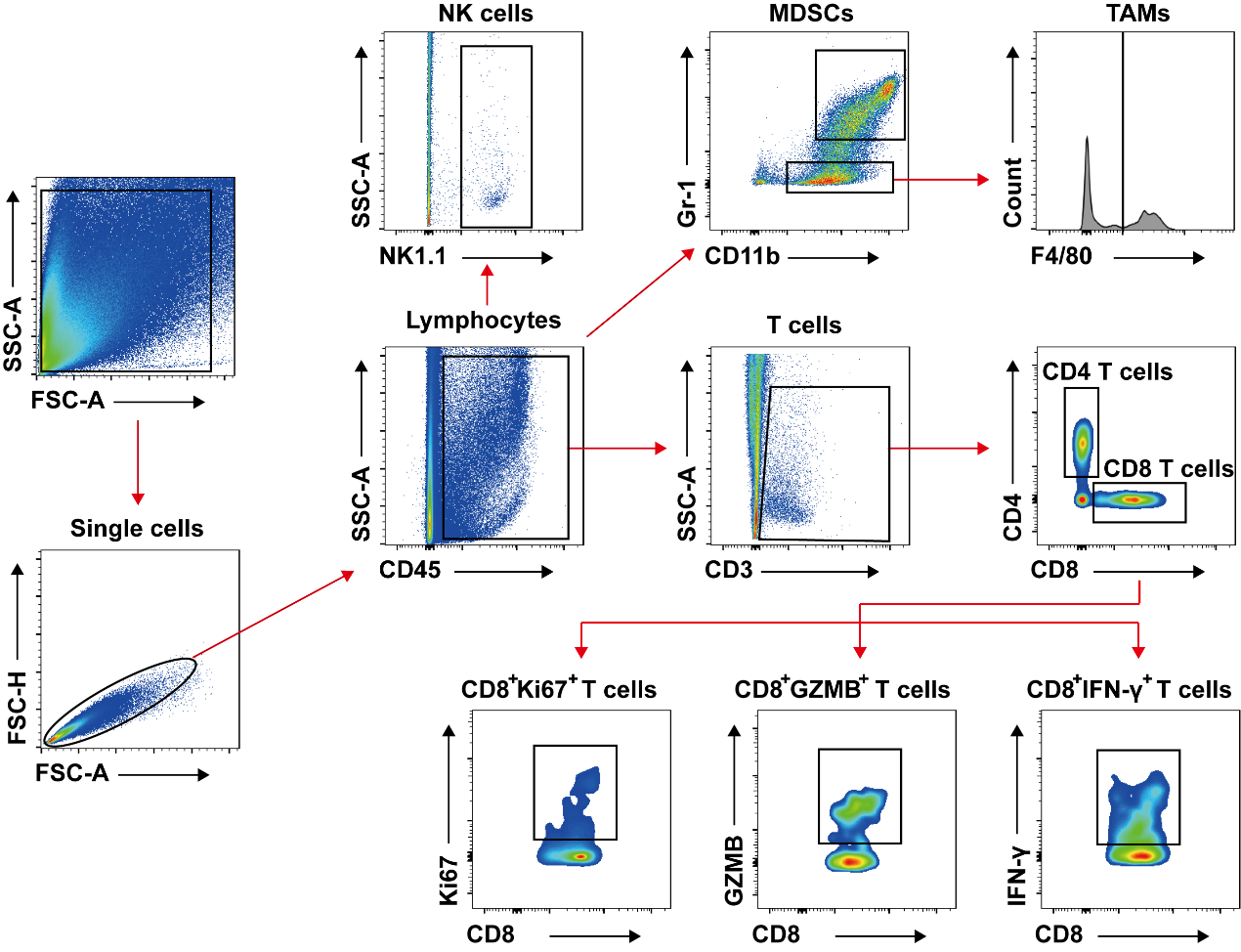


Figure S2. Flow cytometry gating strategy for the analysis of TME immune landscape.


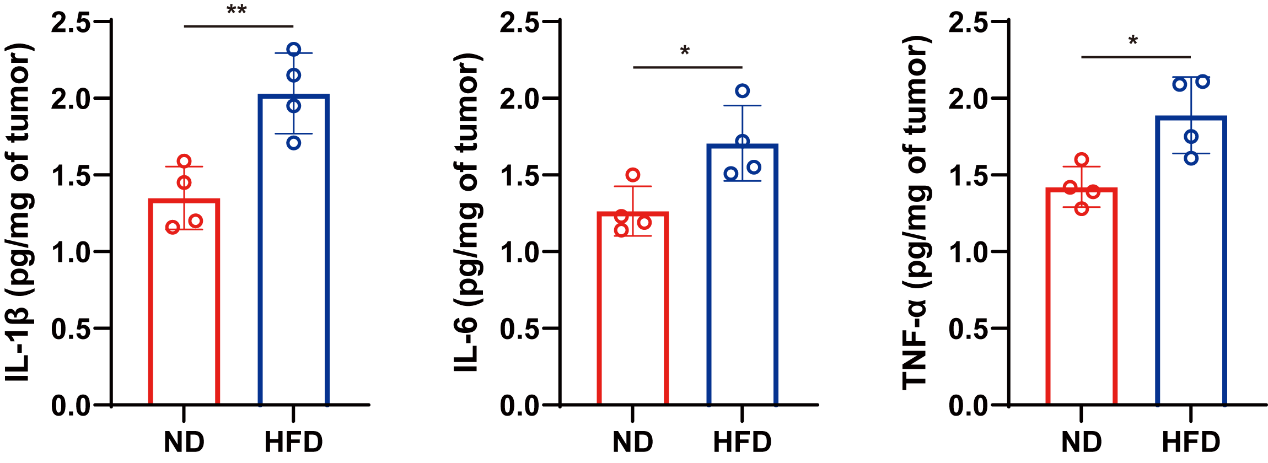


Figure S3. Cytokine levels of IL-1β, IL-6 and TNF-α in tumors of ND or HFD feed mice (n = 4).


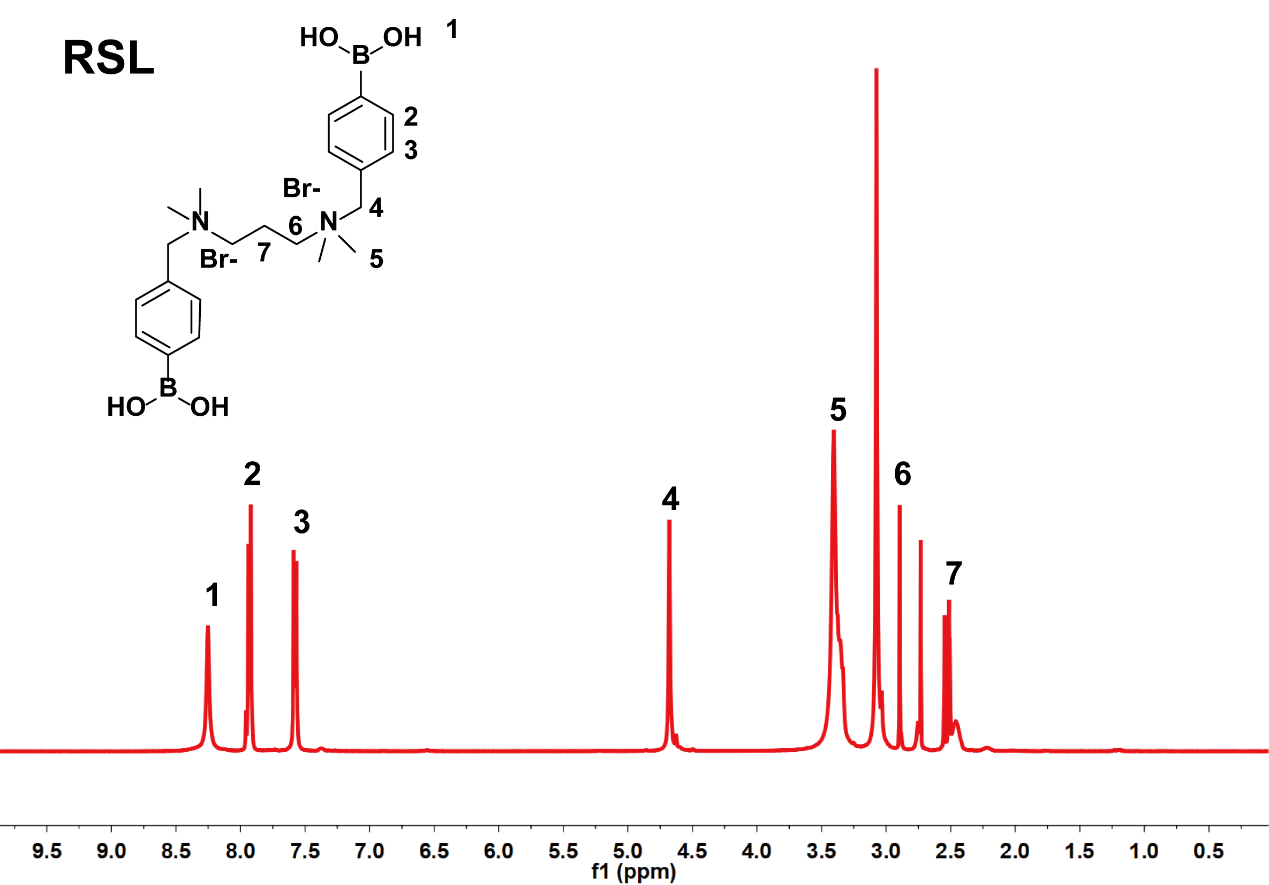


Figure S4. ^1^H-NMR showing the formation of the RSL.


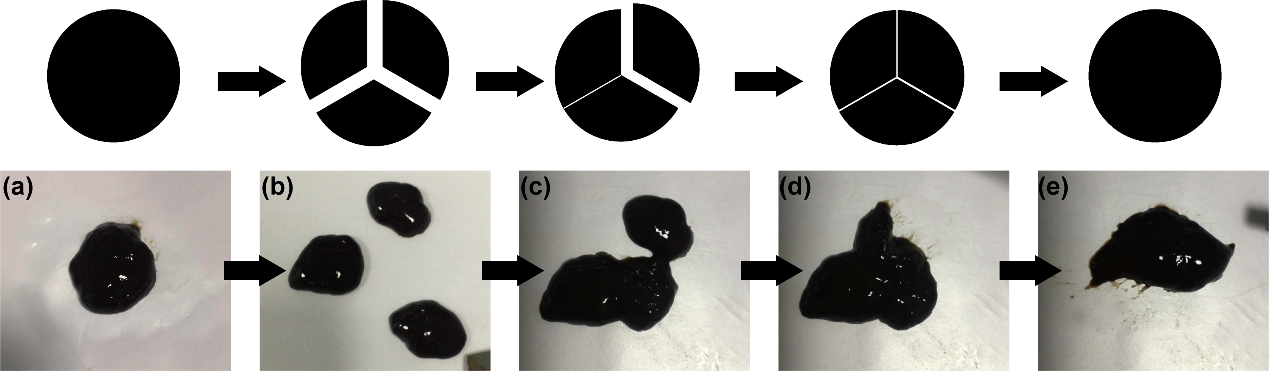


Figure S5. The self-healing property of the hydrogel after cutting.


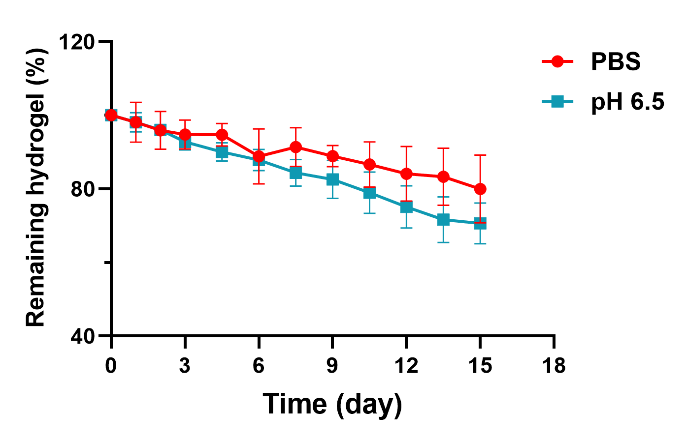


Figure S6. Degradation profile of Lipo/CXB@Hydrogel in blank PBS and pH 6.5 buffer (n = 3).


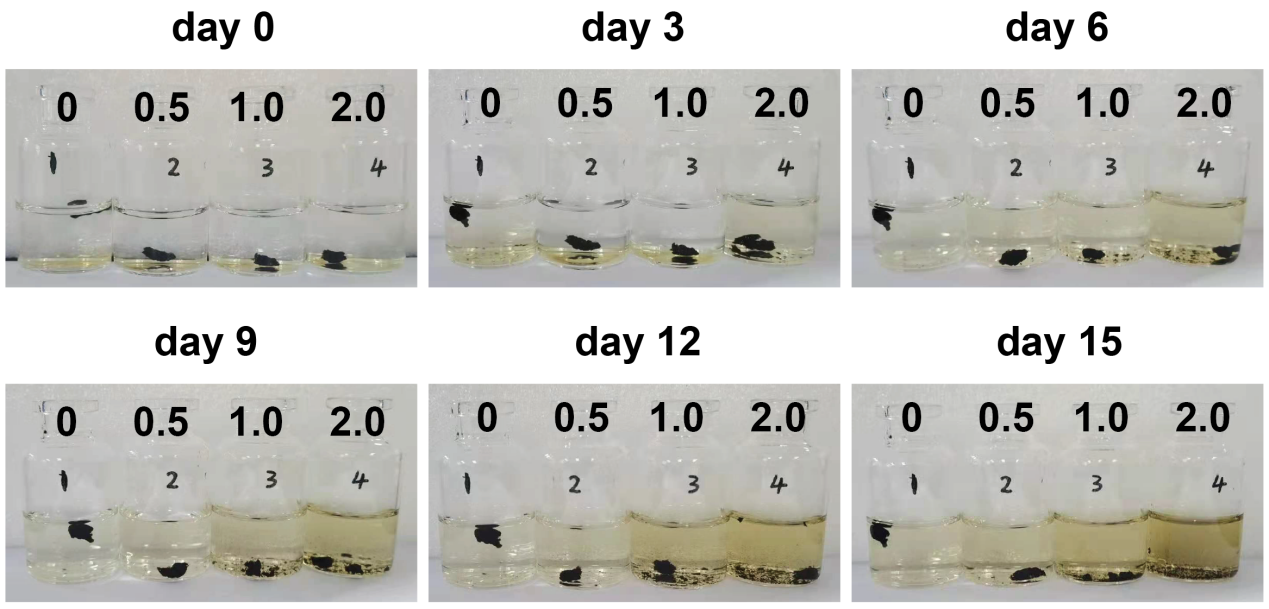


Figure S7. Representative gross images of Lipo/CXB@Hydrogel with various concentration of H_2_O_2_ (0, 0.5, 1.0 and 2.0 mM) incubation at predetermined time points.


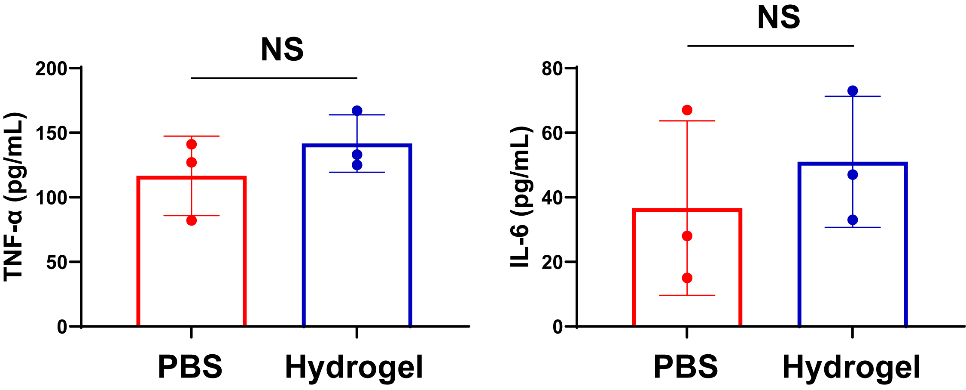


Figure S8. The inflammatory cytokine release from macrophages stimulated by the ROS-degraded hydrogel fragments (n = 3).


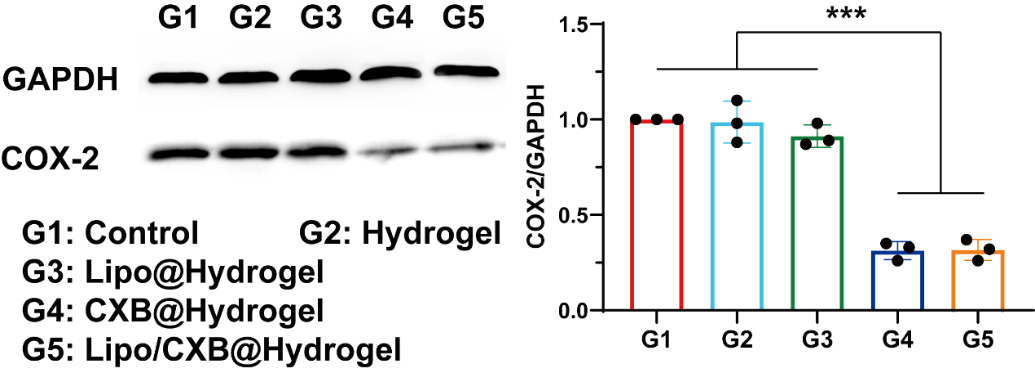


Figure S9. Western blotting showing inhibition of COX-2 in tumor tissues after Lipo/CXB@Hydrogel treatment (n = 3).


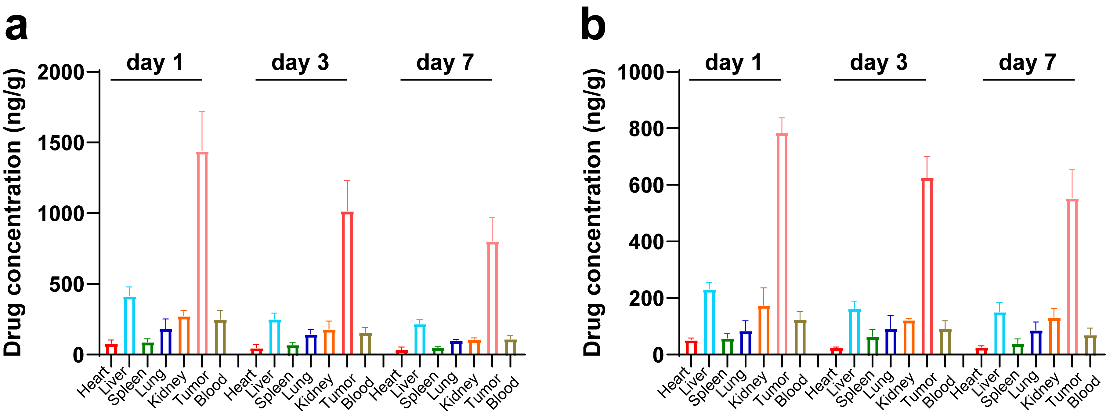


Figure S10. The concentration of (a) CXB and (b) Lipo in tumor, major organs and blood was analyzed on day 1, 3 and 7 after local implantation of Lipo/CXB@Hydrogel (n = 3).


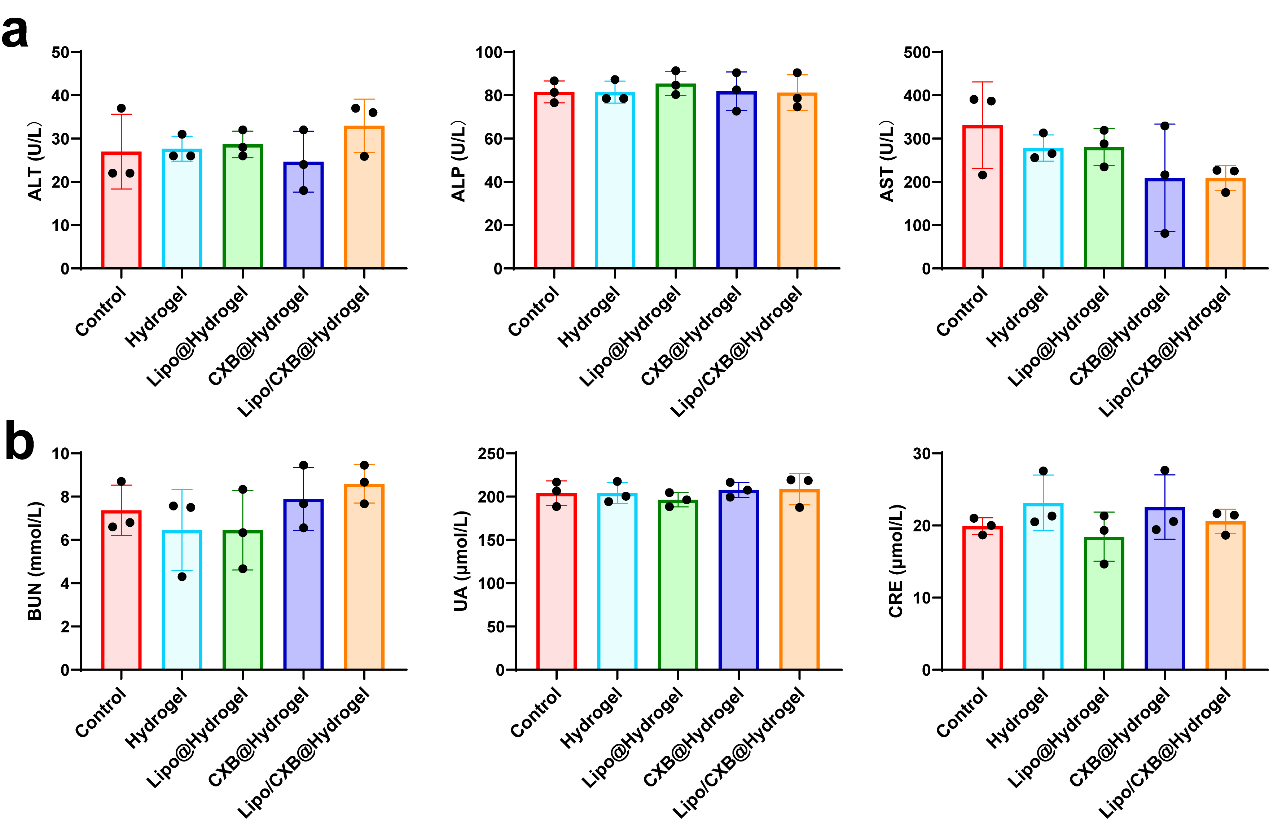


Figure S11. The levels of liver function indicators (a), including ALT, ALP, AST and renal function markers (b), including BUN, UA, CRE from HFD C57/BL6 mice in different groups (Control, Hydrogel, Lipo@Hydrogel, CXB@Hydrogel, and Lipo/CXB@Hydrogel) (n = 3).


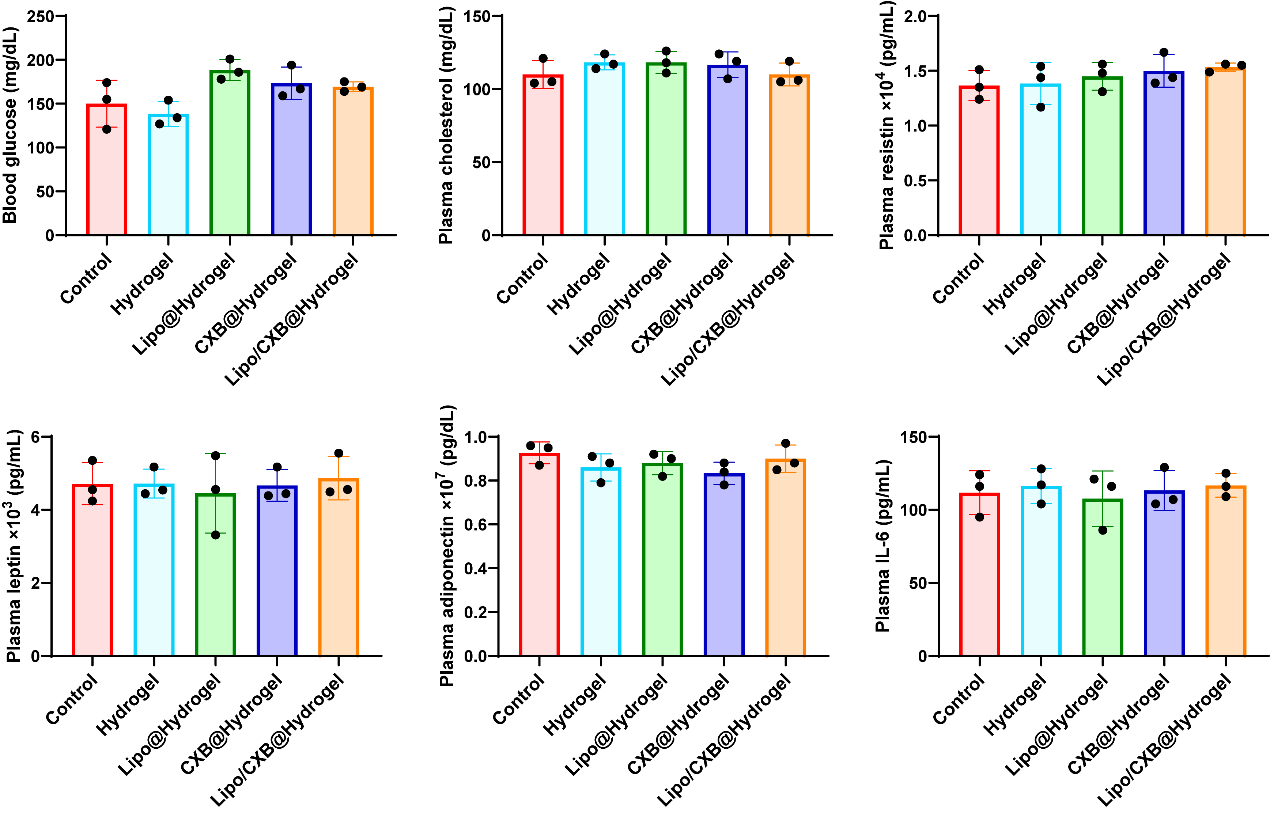


Figure S12. Systemic metabolic and inflammatory parameters including glucose, cholesterol, resistin, leptin, adiponectin and IL-6 from HFD C57/BL6 mice in different groups (Control, Hydrogel, Lipo@Hydrogel, CXB@Hydrogel, and Lipo/CXB@Hydrogel) (n = 3).


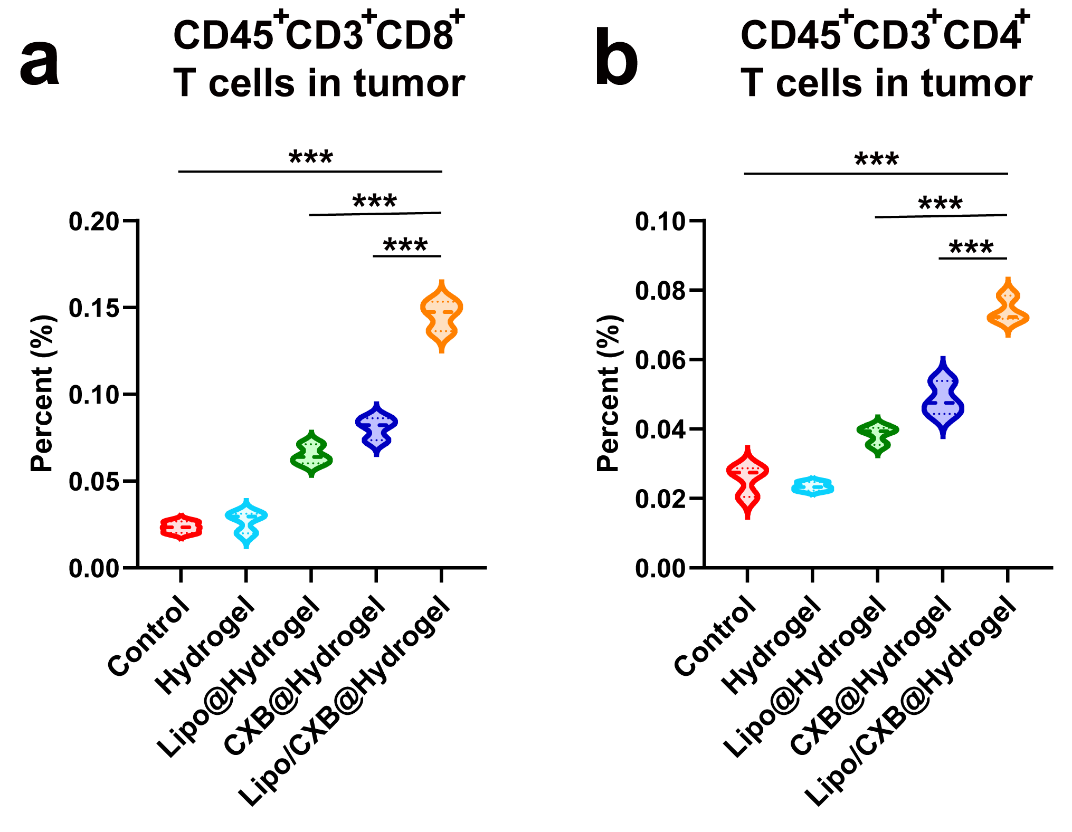


Figure S13. Percentages of CD3^+^CD8^+^ and (b) CD3^+^CD4^+^ T cells in tumors after treatments with PBS, Hydrogel, Lipo@Hydrogel, CXB@Hydrogel and Lipo/CXB@Hydrogel (n = 3).


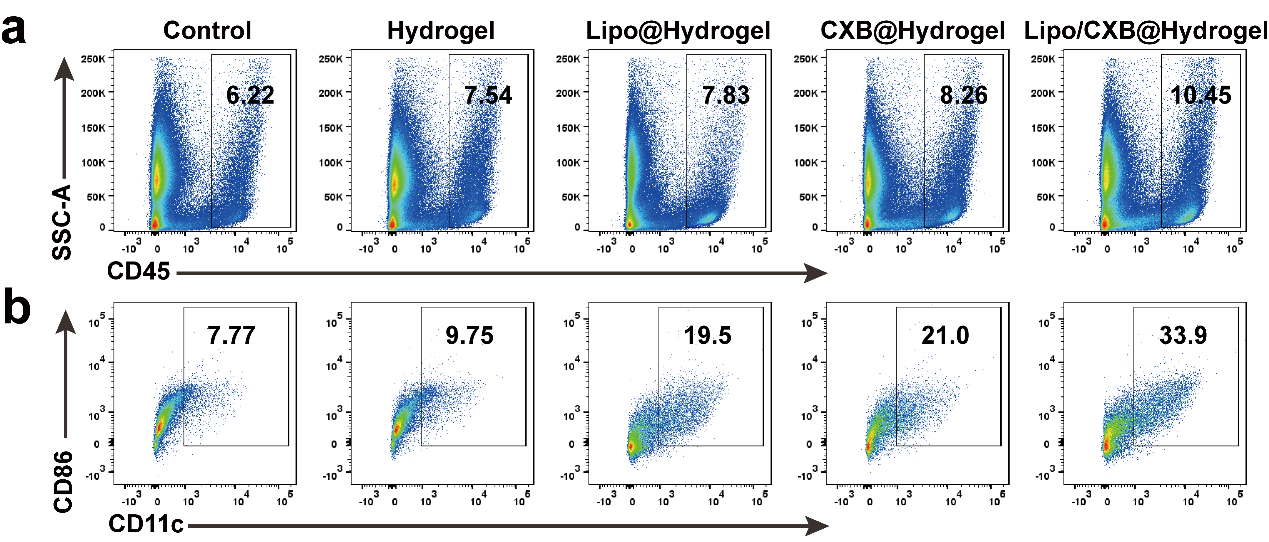


Figure S14. Flow cytometry of (a) lymphocytes (CD45^+^) and (b) matured DCs (CD45^+^CD11c^+^CD86^+^) in tumor after treatments with PBS, Hydrogel, Lipo@Hydrogel, CXB@Hydrogel and Lipo/CXB@Hydrogel.


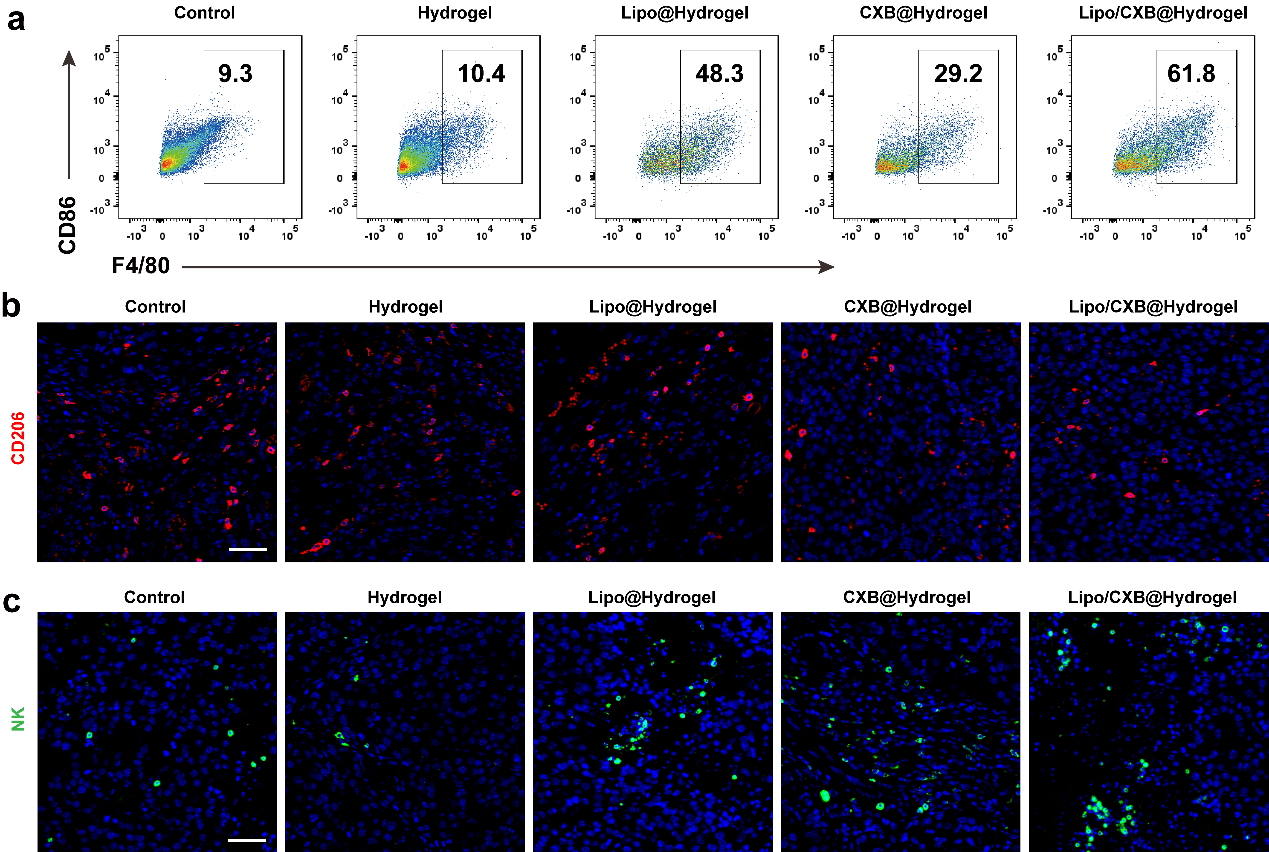


Figure S15. (a) Flow cytometry of M1 TAMs (CD45+CD11b+F4/80+CD86+) in tumor after treatments with PBS, Hydrogel, Lipo@Hydrogel, CXB@Hydrogel and Lipo/CXB@Hydrogel. (b) Immunofluorescence staining of M2 TAMs in tumor after treatments with PBS, Hydrogel, Lipo@Hydrogel, CXB@Hydrogel and Lipo/CXB@Hydrogel. Nuclei: blue, CD206: red. Scale bar = 50 μm. (c) Immunofluorescence staining of NK cells in tumor after treatments with PBS, Hydrogel, Lipo@Hydrogel, CXB@Hydrogel and Lipo/CXB@Hydrogel. Nuclei: blue, NK: green. Scale bar = 50 μm.


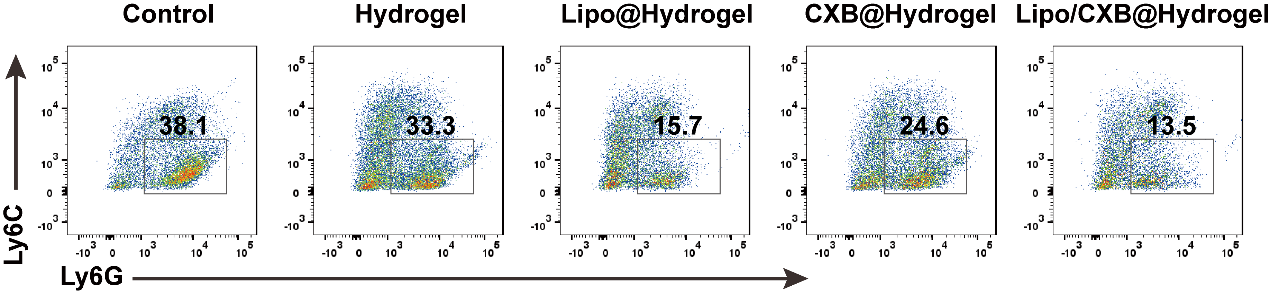


Figure S16. Flow cytometry of PMN-MDCSs (CD45^+^CD11b^+^Ly6G^+^Ly6C^-^) in tumor after treatments with PBS, Hydrogel, Lipo@Hydrogel, CXB@Hydrogel and Lipo/CXB@Hydrogel.


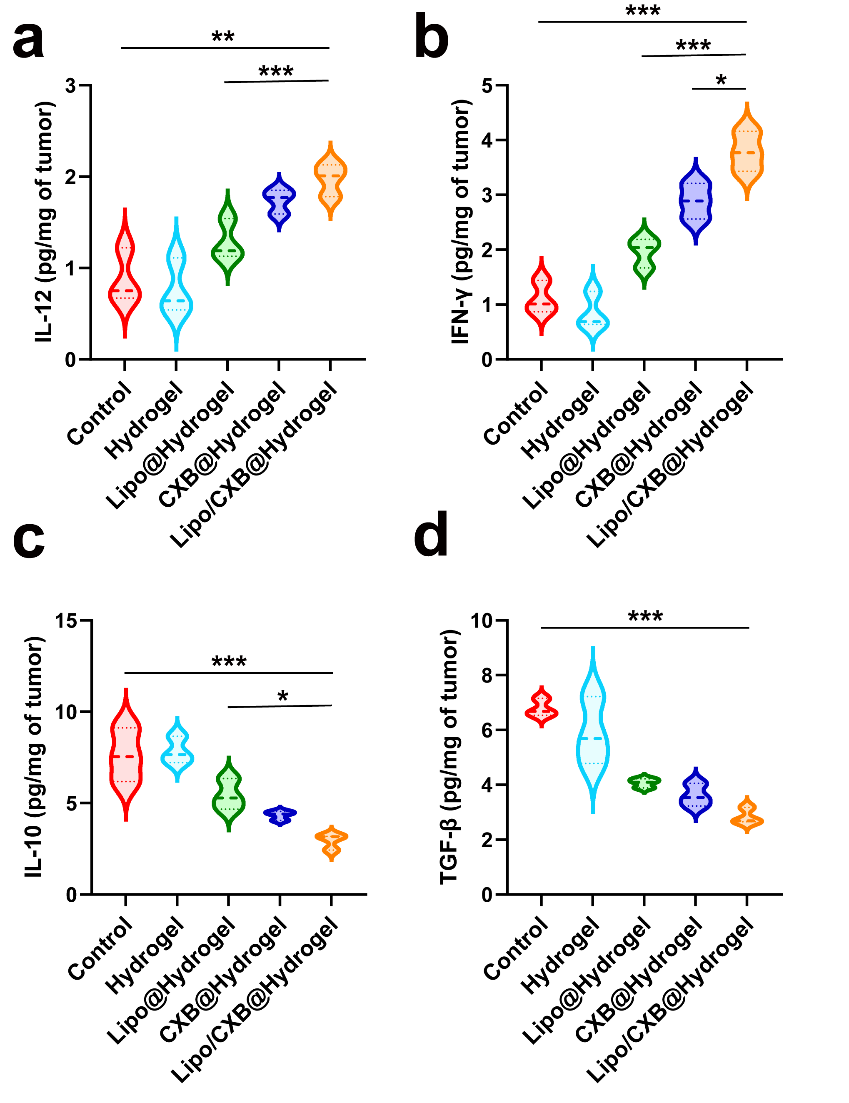


Figure S17. Cytokine levels of (a) IL-12, (b) IFN-γ, (c) IL-10 and (d) TGF-β in tumors after treatments with PBS, Hydrogel, Lipo@Hydrogel, CXB@Hydrogel and Lipo/CXB@Hydrogel (n = 3).


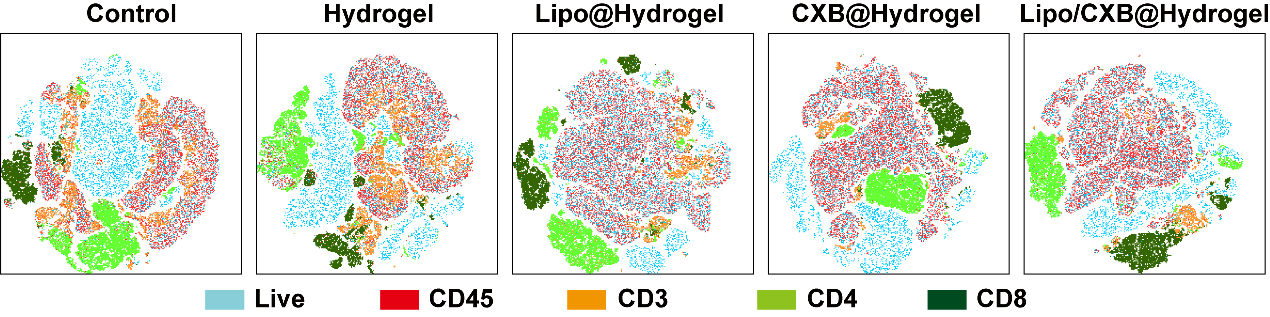


Figure S18. t-SNE analysis of T cell subpopulations in spleen treatments with PBS, Hydrogel, Lipo@Hydrogel, CXB@Hydrogel and Lipo/CXB@Hydrogel.


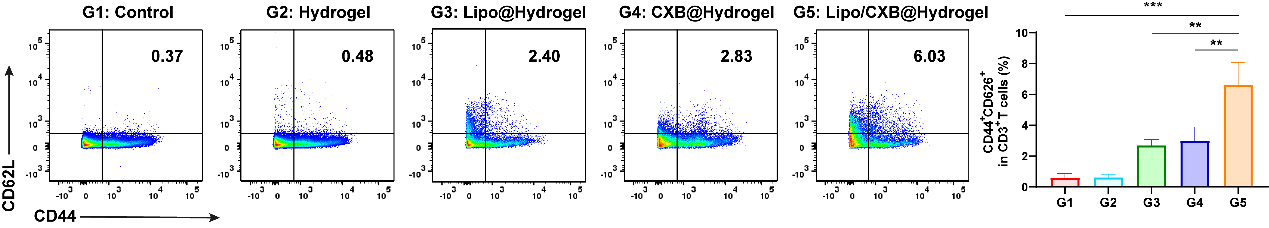


Figure S19. Flow cytometric and quantative analysis of memory T cells (CD45^+^CD3^+^CD44^+^CD62L^+^) in spleen after treatments with PBS, Hydrogel, Lipo@Hydrogel, CXB@Hydrogel and Lipo/CXB@Hydrogel (n = 3).


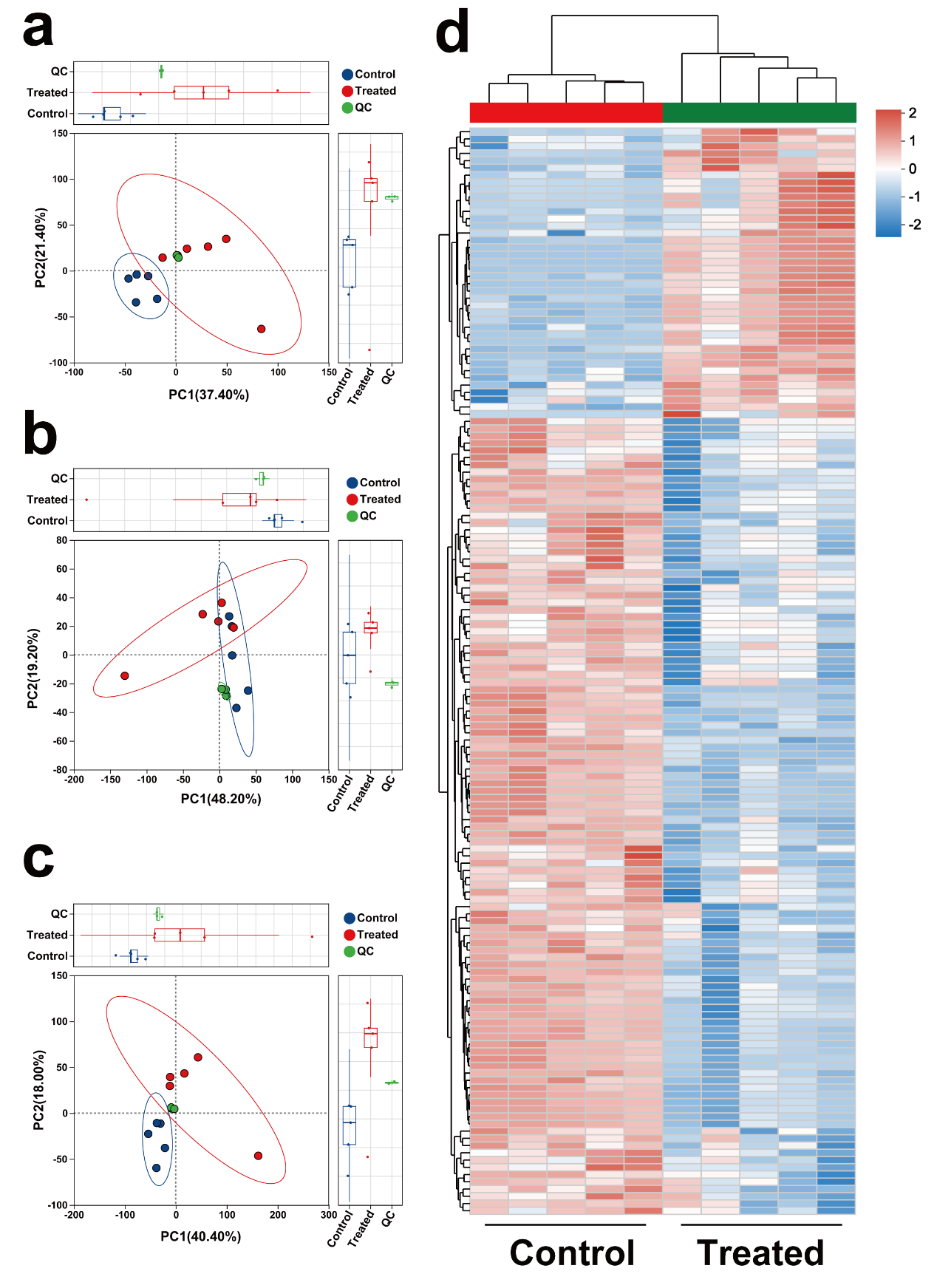


Figure S20. PCA analysis of all expressed metabolites in (a) positive, (b) negative and (c) mixed ionization mode (n = 5). (d) Heat map of the representative differentially metabolites in HFD tumors after PBS and Lipo/CXB@Hydrogel treatments (n = 5).


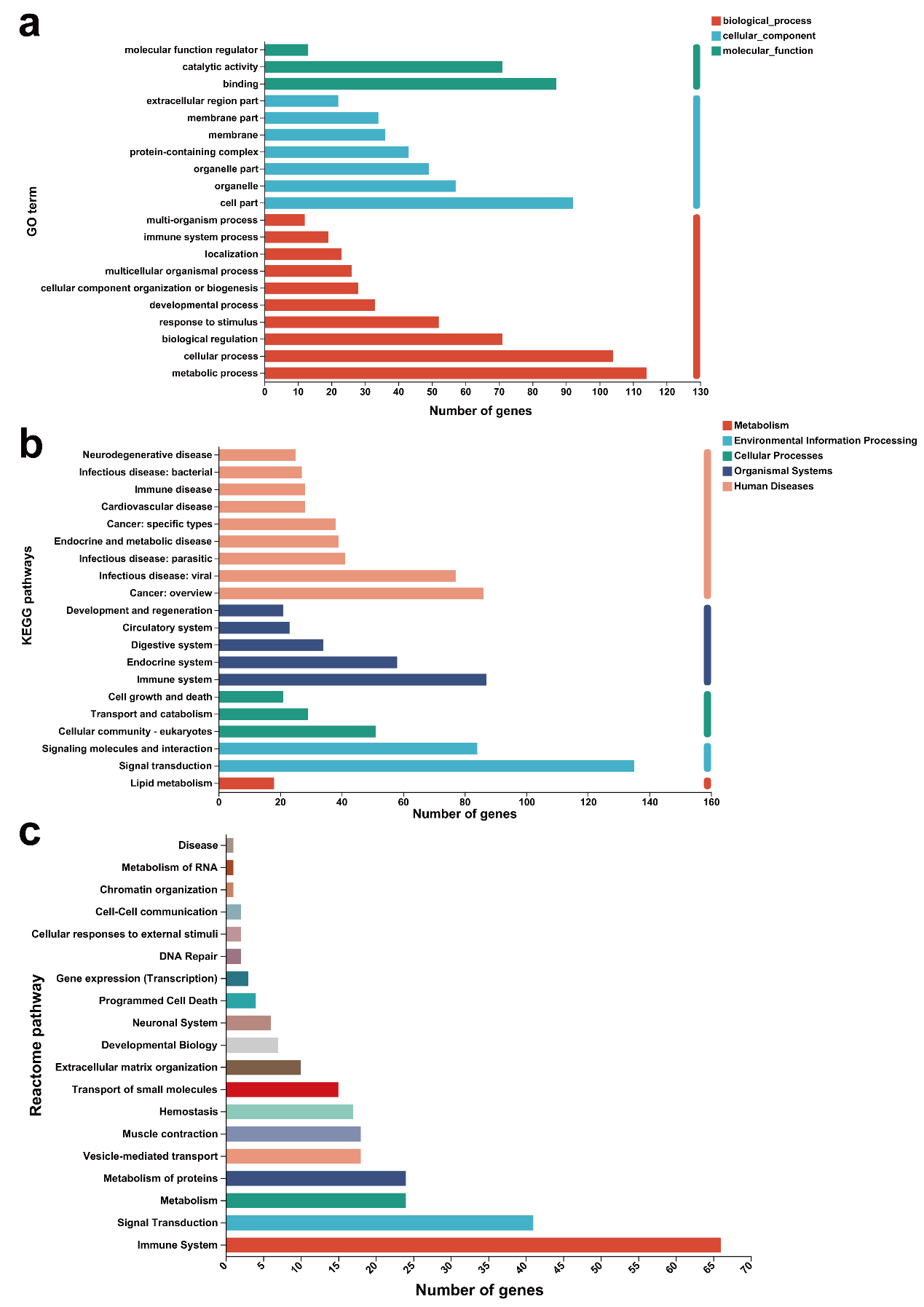


Figure S21. (a) GO annotations, (b) KEGG annotations and (c) Reactome annotations of the differentially regulated genes between untreated and Lipo/CXB@Hydrogel group (n = 3).


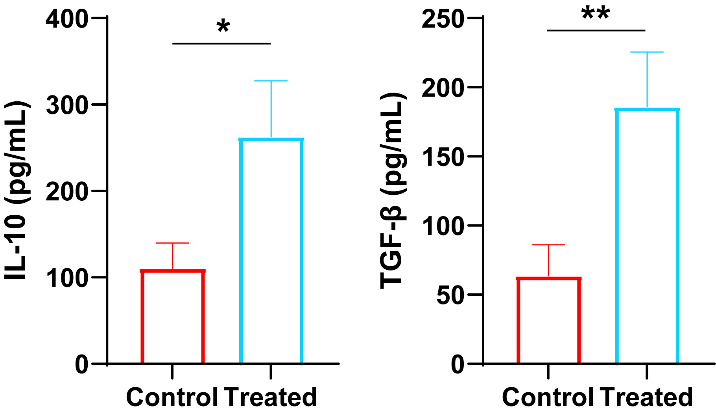


Figure S22. IL-10 and TGF-β production around wound on day 6 in all groups (n = 3).
